# A dual-acting DNASE1/DNASE1L3 biologic prevents autoimmunity and death in genetic and induced lupus models

**DOI:** 10.1172/jci.insight.177003

**Published:** 2024-06-18

**Authors:** Paul R. Stabach, Dominique Sims, Eduardo Gomez-Bañuelos, Sandra Zehentmeier, Kris Dammen-Brower, Andrew Bernhisel, Sophia Kujawski, Sam G. Lopez, Michelle Petri, Daniel W. Goldman, Ethan R. Lester, Quan Le, Tayyaba Ishaq, Hana Kim, Shivani Srivastava, Deepika Kumar, Joao P. Pereira, Kevin J. Yarema, Fotios Koumpouras, Felipe Andrade, Demetrios T. Braddock

**Affiliations:** 1 Department of Pathology, Yale University School of Medicine, New Haven, Connecticut, USA.; 2 Division of Rheumatology, Johns Hopkins University School of Medicine, Baltimore, Maryland, USA.; 3 Department of Immunobiology and Yale Stem Cell Center, Yale University School of Medicine, New Haven, Connecticut, USA.; 4 Translational Tissue Engineering Center and Department of Biomedical Engineering, Johns Hopkins University, Baltimore, Maryland, USA.; 5 Department of Rheumatology, Yale University School of Medicine, New Haven, Connecticut, USA.

**Keywords:** Autoimmunity, Therapeutics, Innate immunity, Lupus, Vasculitis

## Abstract

A defining feature of systemic lupus erythematosus (SLE) is loss of tolerance to self-DNA, and deficiency of DNASE1L3, the main enzyme responsible for chromatin degradation in blood, is also associated with SLE. This association can be found in an ultrarare population of pediatric patients with DNASE1L3 deficiency who develop SLE, adult patients with loss-of-function variants of DNASE1L3 who are at a higher risk for SLE, and patients with sporadic SLE who have neutralizing autoantibodies against DNASE1L3. To mitigate the pathogenic effects of inherited and acquired DNASE1L3 deficiencies, we engineered a long-acting enzyme biologic with dual DNASE1/DNASE1L3 activity that is resistant to DNASE1 and DNASE1L3 inhibitors. Notably, we found that the biologic prevented the development of lupus in *Dnase1^–/–^Dnase1L3^–/–^* double-knockout mice and rescued animals from death in pristane-induced lupus. Finally, we confirmed that the human isoform of the enzyme biologic was not recognized by autoantibodies in SLE and efficiently degraded genomic and mitochondrial cell–free DNA, as well as microparticle DNA, in SLE plasma. Our findings suggest that autoimmune diseases characterized by aberrant DNA accumulation, such as SLE, can be effectively treated with a replacement DNASE tailored to bypass pathogenic mechanisms, both genetic and acquired, that restrict DNASE1L3 activity.

## Abstract 2

An enzyme biologic with dual-acting DNASE1/DNASE1L3 activity prevents autoimmunity and death in lupus models, with implications for other diseases characterized by aberrant DNA accumulation.

## Graphical abstract

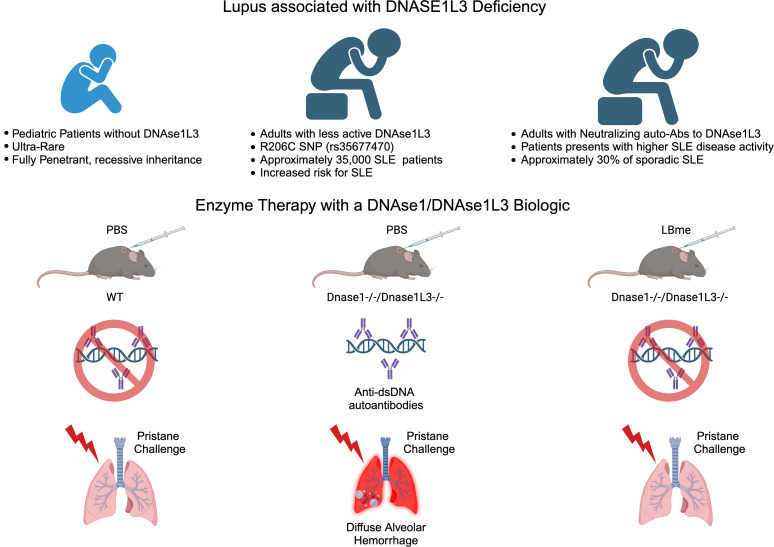

## Introduction

Autoimmune disease is among the 10 leading causes of death in females, and systemic lupus erythematosus (SLE) is one of the five leading causes of death in Black and Hispanic females aged 15–24 years in the United States (1). A common clinical finding in SLE is loss of tolerance to self-DNA, in which patients develop high-affinity pathogenic autoantibodies against histone-bound DNA, antinuclear Abs (ANAs), and anti–double-stranded DNA Abs (anti-dsDNA Abs) (2–4). Anti-dsDNA Abs correlate with disease flares, hypocomplementemia, and lupus nephritis and encompass a heterogenous pool of autoantibodies with distinct physicochemical properties. Autoantibodies that cross-react with ds-DNA and other self-antigens are among the most pathogenic and include those that also recognize α-actinin to mediate lupus nephritis (5, 6), those that cross-react with the N-methyl-D-aspartic acid receptor to increase risk of lupus neuropsychosis (7), and those that neutralize DNASE1L3 and are found in patients presenting with higher disease activity scores (8).

*DNASE1L3* codes for an extracellular enzyme that digests chromatin and microparticle (MP) DNA in the blood. About a decade ago monogenic forms of juvenile SLE were described in children with mutations in *DNASE1L3* that eliminated functional enzyme from the circulation (9–12). This ultra-rare juvenile SLE population, described in 40 patients in the literature, phenocopies idiopathic SLE, and SLE is transmitted in a strictly Mendelian and fully penetrant recessive inheritance pattern. Soon thereafter, a second population of patients with a pathogenic variant of *DNASE1L3* that reduced plasma enzyme concentration by about 80% — R206C (rs35677470) — was found in thousands of patients with lupus, rheumatoid arthritis, and scleroderma, an association that places individuals with this variant at risk for autoimmune disease (13–19). Finally, patients with neutralizing autoantibodies against DNASE1L3 that also bind with high affinity to dsDNA were recently identified in about 30% of patients with SLE (20), and these patients were found to present with more aggressive disease, characterized by higher SELENA-SLEDAI scores at presentation and activated interferon-stimulated gene and neutrophil activation gene cluster signatures (8).

The association of autoimmune disease with DNASE1L3 deficiency, whether from pathogenic variants inducing absent or reduced enzyme concentrations or due to autoantibodies diminishing enzyme activity, implicates DNASE1L3 deficiency as a pathogenic driver of disease phenotype and suggests enzyme replacement as an effective therapeutic strategy in these patients. The presence of neutralizing autoantibodies against DNASE1L3, however, is a significant barrier to using this enzyme as a replacement therapy. Using DNASE1, an abundant enzyme responsible for degrading free DNA in circulation (21), is also not an alternative for treating SLE. Neutralizing Abs against DNASE1 are also common in SLE (22), and the enzyme is ineffective at degrading chromatin (21), which likely explains why DNASE1 did not benefit patients with SLE in a clinical trial (23). To circumvent these limitations, we engineered DNASE1L3 activity onto a DNASE1 enzyme backbone, resulting in a potent DNASE with dual DNASE1 and DNASE1L3 activity that is resistant to inhibitors of both DNASE1 and DNASE1L3. We then tested the efficacy of the engineered biologic (referred to herein as LBme [Lead Biologic, Mouse Equivalent]) in genetic and induced mouse models of lupus.

We quantified the formation of pathogenic anti-dsDNA Abs, ANAs, and anti-histone Abs in mice lacking DNASE1L3 and DNASE1, whose autoimmune disease recapitulates human lupus (24–26), and found that pathogenic autoantibodies could be suppressed by weekly doses of LBme as long as dosing was maintained (up to a year) and that death could be prevented following acceleration of the disease phenotype with pristane. In a (nongenetic) pristane-induced murine model of lupus (27–29), we found that the effects were even more dramatic — LBme rescued animals from death using a therapeutic dosing strategy in 2 strains of C57BL/6 mice. Finally, to confirm that our biologic was active in plasma from patients with lupus, we showed that the human isoform of the engineered biologic degraded cell-free (cf) genomic DNA (cf-gDNA), cf mitochondrial DNA (cf-mtDNA), MP-gDNA, and MP mitochondrial DNA (MP-mtDNA) in the plasma of patients with lupus and was nonreactive to neutralizing DNASE1L3 and DNASE1 autoantibodies present in the plasma of these patients. Our findings have important implications for the treatment of patients with autoimmune diseases associated with genetic and acquired DNASE1L3 deficiency.

## Results

### Dnase1^–/–^Dnase1L3^–/–^ double-knockout mice.

**Figure 1:**
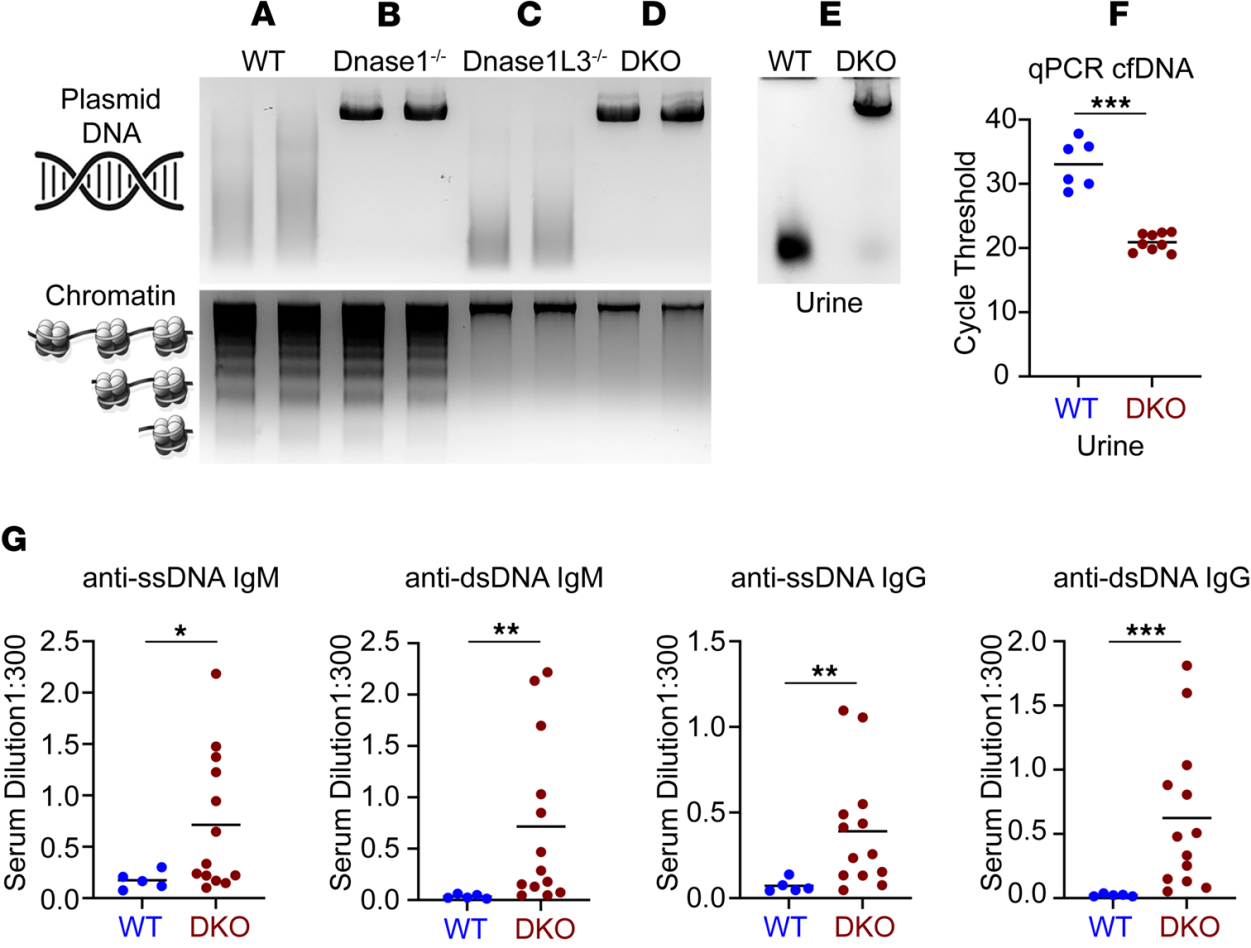
Characterization of WT, *Dnase1^–/–^*, *Dnase1L3^–/–^,* and *Dnase1^–/–^Dnase1L3^–/–^* mice. (**A**–**D**) Plasma activity from 2 representative mice from each genotype (WT, *Dnase1^–/–^*, *Dnase1L3^–/–^,* and *Dnase1^–/–^Dnase1L3^–/–^* [DKO]) against free and chromatin DNA. (**A**) Plasma from WT mice digests exogenously added plasmid DNA into a smear when imaged on a 1% agarose gel (top) and into a ladder pattern resulting from internucleosome cleavage when incubated with chromatin DNA (bottom). (**B**) Plasma from *Dnase1^–/–^* mice cannot digest exogenously added free DNA but does digest chromatin DNA. (**C**) Plasma from *Dnase1L3^–/–^* mice cannot digest chromatin DNA but does digest free DNA. (**D**) Plasma from DKO mice cannot digest either chromatin or free DNA. (**E** and **F**) Urine activity of WT and DKO mice against free DNA. (**E**) Degradation of plasmid DNA added to the urine of WT and DKO mice demonstrates the absence of urine DNASE1 activity in DKO mice. (**F**) Analysis of urine cfDNA by qPCR shows a significant decrease in the Ct in DKO mice, demonstrating increased cfDNA concentrations in DKO mouse urine. (**G**) Appearance of autoantibodies against ssDNA and dsDNA in 8-week-old DKO mice. DKO mice were found to significantly elevate autoantibodies against ssDNA and dsDNA by 8 weeks of age. *P < 0.05, **P < 0.01, ***P < 0.001, 2-tailed Student’s *t* test.

We crossed *Dnase1L3^–/–^* mice with *Dnase1^–/–^* mice to generate *Dnase1^–/–^Dnase1L3^–/–^* double-knockout (DKO) mice on a C57BL/6 background to develop a genetic lupus murine model lacking both DNASE1 and DNASE1L3. To characterize the plasma DNA degrading activity in our in vivo system, we incubated exogenous free DNA or chromatin with sera taken from *Dnase1^–/–^*, *Dnase1L3^–/–^*, and DKO mice for 5 minutes at 37°C and ran the reactions on agarose gels (Figure 1). The sera of WT mice digests free DNA into a smear and chromatin into an internucleosomal ladder separated by about 150 base pairs (Figure 1A). The sera of *Dnase1^–/–^* mice cannot hydrolyze free DNA but fully digests chromatin into an internucleosomal ladder (Figure 1B). Similarly, the sera of *Dnase1L3^–/–^* mice fully digests the free DNA but is unable to digest the chromatin (which appears as a band at the top of the gel, Figure 1C), and the sera of DKO mice cannot hydrolyze either free DNA or chromatin (Figure 1D). Endogenous differences of free DNA were quantitated in the urine of the mice using quantitative PCR (qPCR), which demonstrated lower Ct for DKO mice than WT mice, consistent with greater urine concentrations of DNA (Figure 1, E and F). The effects of DNASE1/1L3 absence on the adaptive immune system of DKO mice were apparent by 8 weeks, when anti–single-stranded DNA (anti-ssDNA) and anti-dsDNA IgM and IgG Abs were noted to be significantly elevated in comparison to those of WT mice, confirming loss of tolerance to self-DNA similar to what is reported in *Dnase1^–/–^* and *Dnase1L3^–/–^* mice (24, 26) (Figure 1G). Finally, just as plasma from patients with SLE stimulates neutrophil extracellular trap (NET) formation (also called NETosis) in the neutrophils of individuals acting as healthy controls (HCs) (30–32), we found that plasma from DKO mice induced NETosis in WT murine neutrophils over and above the effects of plasma from WT mice (Supplemental Figure 1; supplemental material available online with this article; https://doi.org/10.1172/jci.insight.177003DS1).

### Engineering dual-acting DNASE1/DNASE1L3 enzymes.

We first amplified the genes for *Dnase1* and *Dnase1L3* from a mouse C57BL6/J cDNA library and cloned them into a modified pFUSE-mIgG1-Fc1 plasmid containing the T285Y and T289E amino acid substitutions in the mouse Fc domain (based on GenBank AXV45364.1), in order to enhance the FcRn-mediated endosomal recycling as previously described (33). We began our attempts to engineer a dual-acting enzyme by conferring DNASE1L3 activity onto the DNASE1 scaffold and conversely conferring DNASE1 activity onto the DNASE1L3 scaffold. To do so, we employed three approaches: first, we reviewed the literature regarding hyperactive mutations of human DNASE1 and mutations that block actin inhibition (34–36); second, we scanned the sequence of DNASE1 and DNASE1L3 in nonhuman species for relevant positively charged sequence polymorphisms on the surface of the protein facing the DNA binding domain in order to enhance the affinity of the enzymes for the negatively charged phosphates on the DNA backbone; and third, we scanned DNASE1 and DNASE1L3 in multiple species for surface N-linked glycosylation sequences in preferred locations. We then made a list of possible favorable point mutations in DNASE1 and DNASE1L3 to test and designed 46 pairs of DNA oligonucleotides to incorporate the putative sequences into the backbones of DNASE1 and DNASE1L3 (Supplemental Tables 1–4), keeping in mind that we were attempting to identify the minimum possible number of substitutions to avoid immunorejection of an optimized enzyme biologic.

To evaluate potentially beneficial amino acid substitutions in an efficient manner, we combined several pairs of oligos into a single site-directed mutagenesis reaction and sequenced the resulting clones. An attempt was also made to cut and paste various fragments of DNASE1L3 (with particular emphasis on the unique carboxy terminal tail) into the backbone of DNASE1 to generate chimeric clones with dual activity. In total, 18 DNASE1 and 25 DNASE1L3 murine isoforms (Table 1) and 18 DNASE1 and 9 DNASE1L3 human isoforms (Table 2) were created and screened for DNASE1 and DNASE1L3 activity in the conditioned media of CHO cell clones transiently expressing the enzymatic isoforms. From the results of this study, we concluded that a bifunctional enzyme could be more readily achieved on a DNASE1 backbone than on a DNASE1L3 backbone and thereafter abandoned the development of a bifunctional enzyme on a DNASE1L3 backbone.

### Additional modifications to enhance bioavailability and potency.

**Figure 2:**
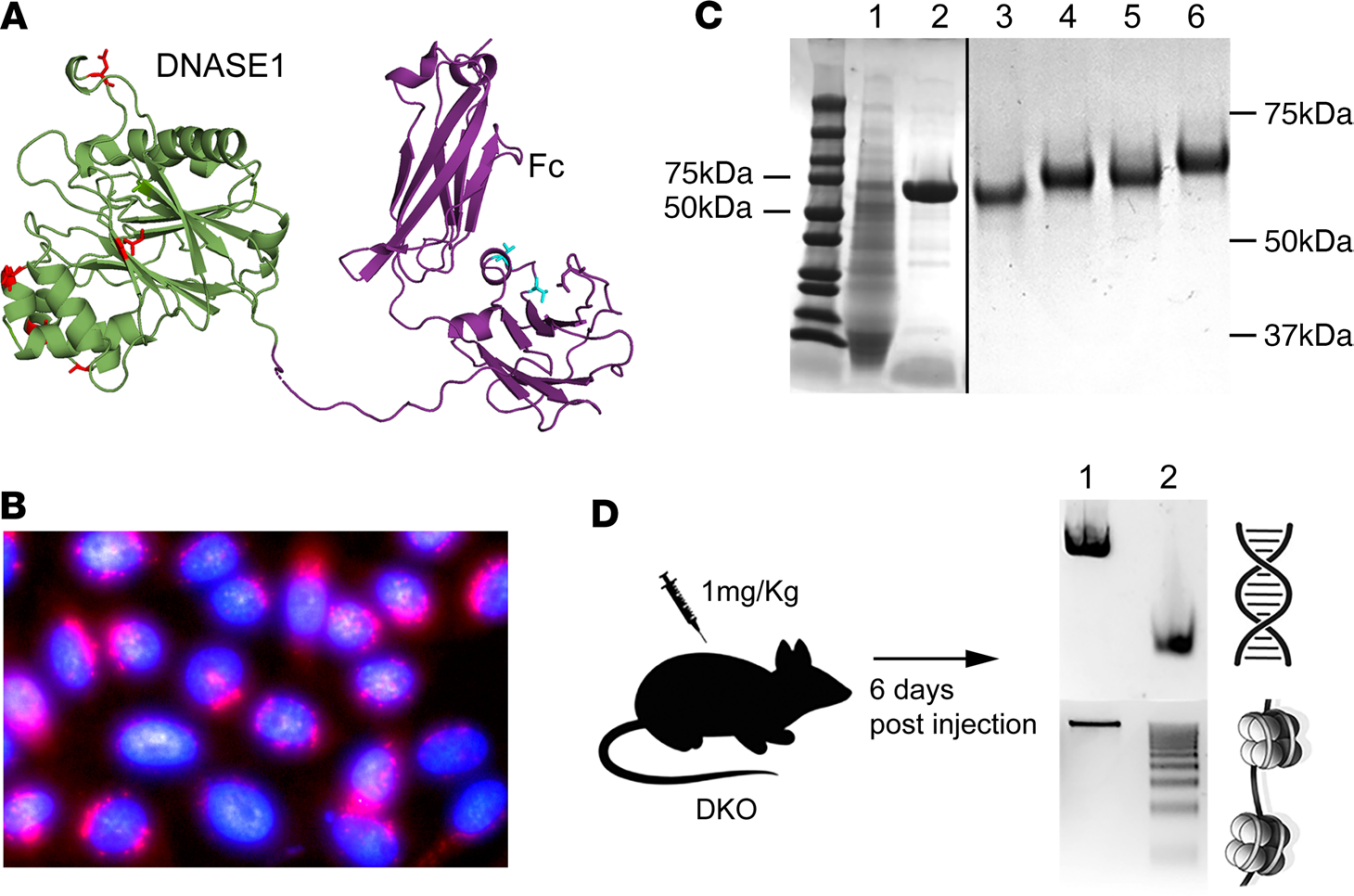
Overview of the design, production, purification, and pharmacodynamics of optimized biologics. (**A**) Isoforms of DNASE1 and DNASE1L3 enzymes (green) with various point mutations (red) chosen to confer dual activity and actin resistance were fused to IgG1 Fc domains, which were further optimized to enhance bioavailability via enhanced FcRn recycling using point mutations (cyan). (**B**) Fluorescent staining of CHO cells stably transfected with human ST6GAL1 to enhance sialylation of the biologic. Red fluorescence highlights ST6GAL1 in a peri-Golgi distribution, and cyan fluorescence highlights nuclei. Original magnification, ×100. The biologics were produced in these CHO cells, and sialic acid precursors were added to the growth media to glycopolish the enzymes. (**C**) SDS-PAGE of enzyme biologics during purification. Lane 1 shows concentrated extracellular CHO cell–conditioned media before affinity chromatography; lane 2 shows purified DNASE1-Fc parent construct 1587 with a predicted MW of 58 kDa; lane 3 shows construct 1671; lanes 4 and 5 represent two different preparations of LBme; and lane 6 shows construct 1689. Newly formed N-glycans in the parent sequence exhibit an increased mobility shift. (**D**) Pharmacodynamic studies were conducted in vivo in DKO mice to establish bioavailability, dose range, and dose frequency. DKO mice were injected with a single s.c. dose of biologic, their serum was sampled at various time points and incubated with free DNA and chromatin for 5 minutes at 37°C, and the digestion of plasma was imaged on an agarose gel. The example provided was taken from serum of a DKO mouse before (lane 1) and 6 days after (lane 2) a single s.c. dose of 1 mg/kg LBme. All dosed mice were confirmed to have similar biologic activity.

To further optimize the bifunctional enzyme isoforms, we next produced the biologics in CHO-K1 cells (ATCC) stably cotransfected with human β-galactoside α-2-6-sialyltransferase (ST6GAL1) and grew the cells in media supplemented with 1,3,4-O-Bu_3_ManNAc to “glycopolish” the biologic to enhance sialic acid incorporation, as previously described (33). The biologics were then purified to homogeneity in endotoxin-free conditions using techniques and conditions developed in our laboratory for ENPP1-Fc (33, 37). Analytical HPLC performed on the samples following purification revealed a single major peak with a small trailing peak (Supplemental Figure 2). Size-exclusion chromatography with light scattering, undertaken to assess aggregation state and hydrodynamic behavior, revealed that the protein biologic eluted as a single peak, with the predicted molecular weight of a stable dimer at two separate concentrations of sample loading, containing a polypeptide of ≈119 kDa with 13 kDa of sugars for a total molecular weight of 131 kDa, and a hydrodynamic radius of 5.3 nm (Supplemental Figure 3). Figure 2 summarizes the workflow and key outcomes of biologic development.

### In vivo pharmacodynamic experiments to determine dosing interval.

**Figure 3:**
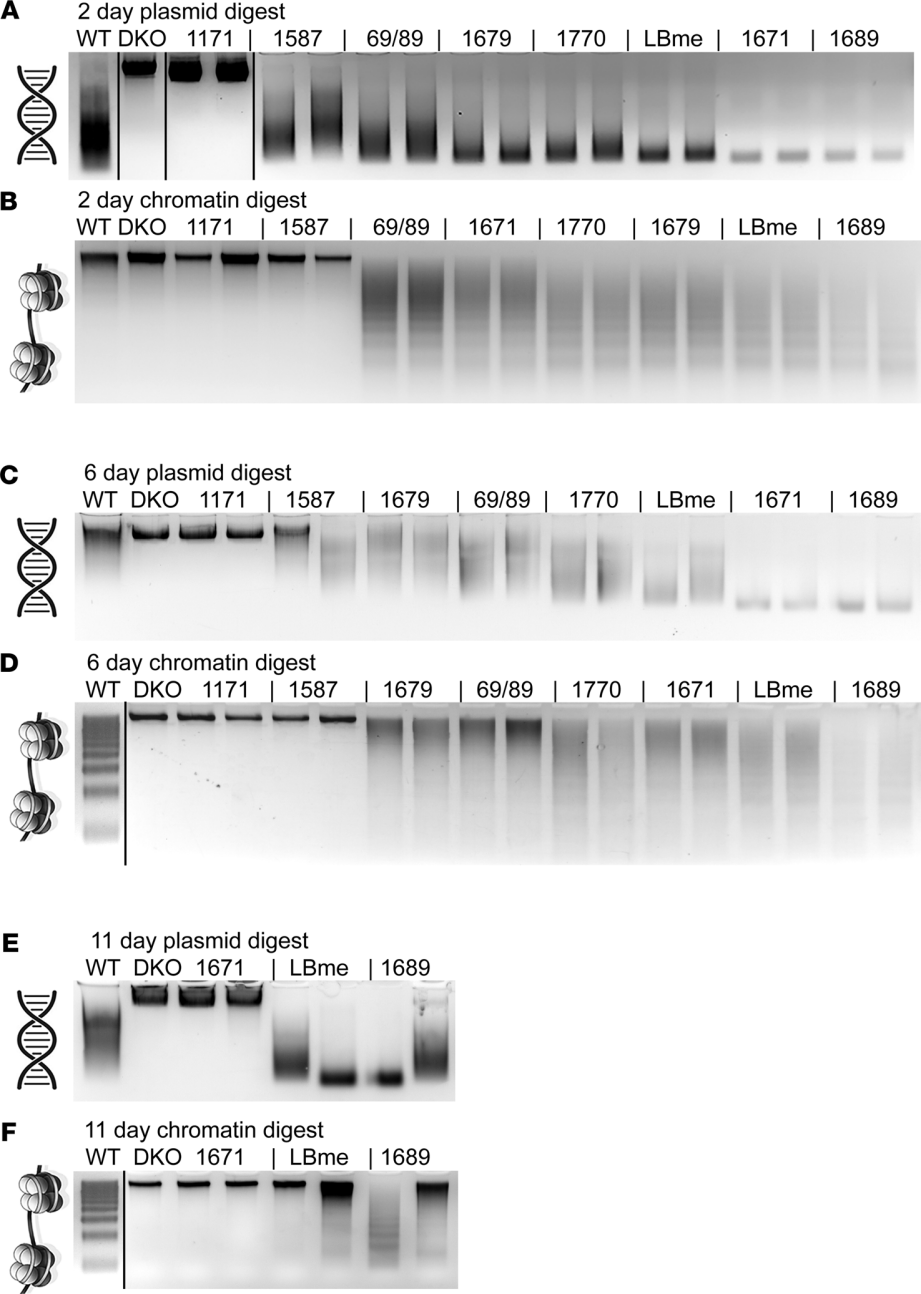
Pharmacodynamic activity of dual activity DNASE1 isoforms. The pharmacodynamic (PD) activity of various purified DNASE1 isoforms was evaluated in vivo by dosing 2 DKO mice for each biologic with a single s.c. injection at 1 mg/kg and withdrawing blood from the mice at 2 (**A** and **B**), 6 (**C** and **D**), and 11 days (**E** and **F**) after dosing. Serum was isolated from the blood samples, and exogenous free plasmid DNA (**A**, **C**, and **E**) and chromatin (**B**, **D**, and **F**) was added. The samples were then incubated at 37°C for 5 minutes and run on agarose gels to visualize degradation of the exogenous DNA (or lack thereof) of each isoform at various time points. Most biologics exhibited full PD activity 2 days after dosing, and 3 isoforms — 1671, 1689, and LBme — exhibited full PD activity 6 days after dosing. Serum from these 3 mice was drawn 11 days after dosing, revealing the murine isoforms with the longest PD activity to be LBme and 1689 (refer to Tables 1 and 2 for clone details). The experiment was performed on two separate occasions.

To determine effective dose levels and frequencies, we analyzed the degradation of exogenous free DNA and chromatin added to sera collected from DKO mice at 2, 6, and 11 days following a single s.c. dose of each enzymatic isoform at a concentration of 1 mg/Kg (Figure 3). The original murine isoform (construct 1171), which lacked an Fc domain due to proteolytic cleavage after purification, exhibited robust activity in vitro but completely lost plasma activity 2 days after injection. The proteolytic cleavage site was identified and resolved in subsequent constructs with a single Arg-to-Gly substitution in the linker domain (Supplemental Figure 4, lanes 1–3). All subsequent full-length enzyme isoforms exhibited potent plasma activity 2 days after dosing, illustrating the profound effect Fc fusions have on biologic half-life in vivo, an attribute we previously exploited to improve the circulation time of ENPP1 (37). With selective enhancement of glycan number and sialic acid content, isoforms LBme and 1689 exhibited full activity in plasma collected 6 days following dosing and partial activity in plasma collected 11 days after dosing (Figure 3). These experiments successfully identified enzymatic isoforms with the greatest bioavailability and established approximate dose levels and frequencies for the in vivo studies.

### Characterization of lead isoforms.

In contrast to commercial DNASE1 (Roche), our lead murine Dnase1-Fc isoform (LBme) hydrolyzed chromatin in a concentration dependent manner (Supplemental Figure 5, A–C) and was resistant to actin inhibition (Supplemental Figure 5D). The internucleosome cleavage of chromatin by LBme and our lead human DNASE1-Fc isoform — 1833 — was not inhibited by heparin or plasmin, known inhibitors of DNASE1L3 (Supplemental Figure 5E) (38). In summary, starting from a DNASE1 backbone, we successfully developed murine and human enzyme biologics with dual DNASE1 and DNASE1L3 activity that are resistant to physiologic inhibitors of DNASE1 or DNASE1L3. The enzyme isoforms exhibited long half-lives and good bioavailability in vivo, permitting weekly dosing intervals at low single-digit mg/Kg levels.

### Prevention of autoimmunity and mortality in genetic models of lupus.

**Figure 4:**
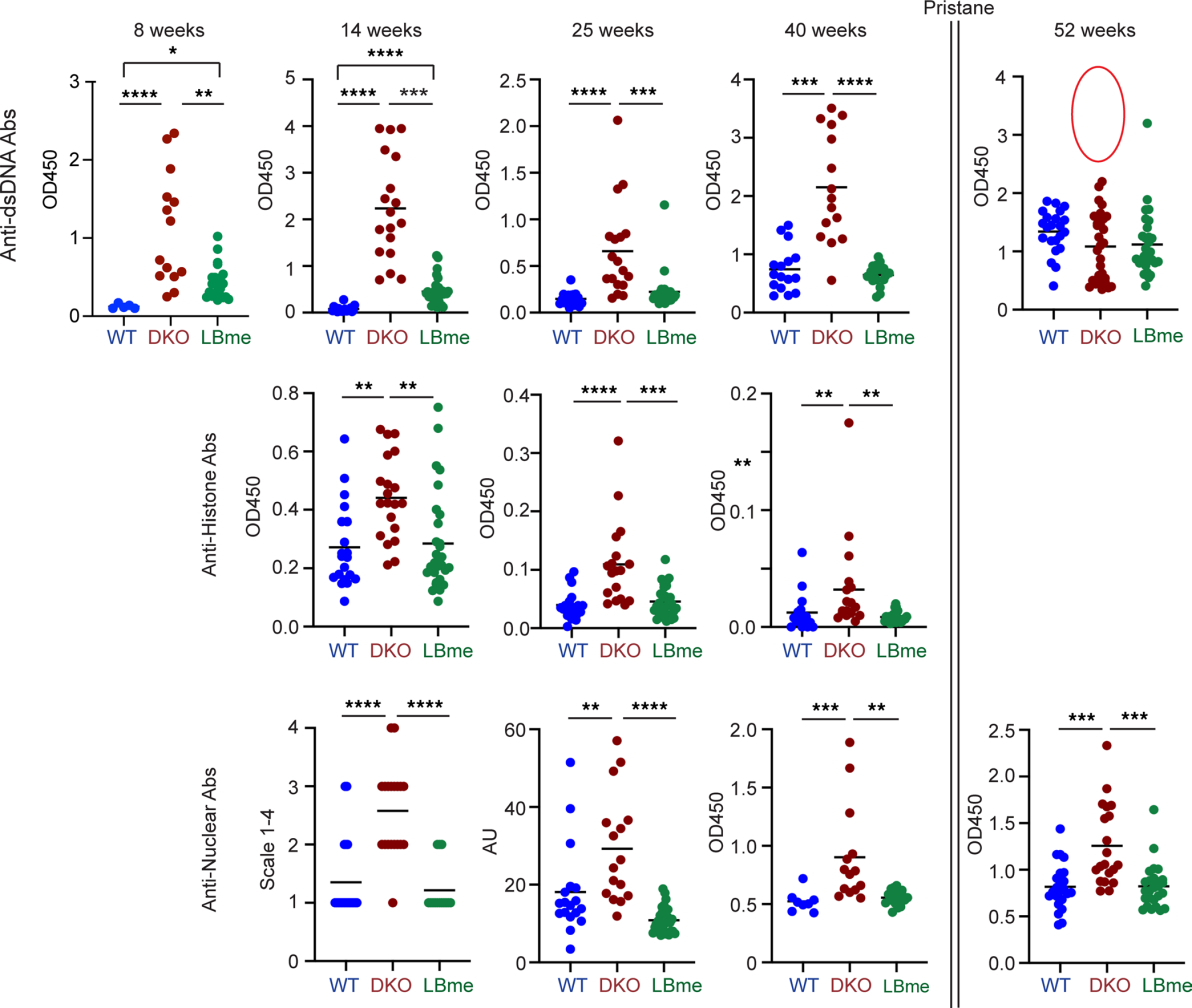
Prevention of autoimmunity in DKO mice by LBme. WT mice were treated with weekly injections of PBS, and DKO mice were treated with weekly s.c. doses of PBS (vehicle) or LBme at 1 mg/kg, from the second week after birth. Serum samples were taken at 8, 14, 25, and 40 weeks, and titers of anti-histone and anti-dsDNA autoantibodies were evaluated by ELISA. Antinuclear Abs at 14 weeks were estimated by visual inspection for signal intensity on a scale from 1 to 4 under a fluorescent microscope. At 25 weeks the intensity was determined from captured fluorescent images using NIH ImageJ software, and at 40 and 52 weeks the intensity of ANA Abs was determined by an ELISA. In comparison to WT controls, by 8 weeks of age the PBS-treated DKO mice demonstrated spontaneously elevated anti-dsDNA and anti-histone and, at 14 weeks, antinuclear Abs, whereas LBme-treated DKO mice did not elevate any autoantibodies suggestive of lupus. At 40 weeks all mice were challenged with pristane (500 μL i.p.), and animals were followed over the next 12 weeks. At 52 weeks the ANAs in the surviving DKO mice remained elevated. However, anti-dsDNA Abs were no longer elevated, demonstrating increased mortality in vehicle-treated DKO mice with elevated anti-dsDNA Ab titers. *P < 0.05, **P < 0.01, ***P < 0.001, ****P < 0.0001, ANOVA Kruskal-Wallis test.

Monogenic murine models of lupus — i.e., *Dnase1^–/–^* or *Dnase1L3^–/–^* mice — faithfully reproduce the lupus phenotype present in humans, including elevated titers of autoantibodies against dsDNA, histones, and chromatin (ANAs), splenomegaly with expanded white pulp (germinal center B cells); and background-dependent glomerulonephritis, which appears in a 129 inbred mouse background (24–26). To test whether autoimmunity in genetic murine models could be prevented by our enzyme biologic, we dosed DKO mice weekly with 1 mg/kg s.c. injections of LBme and followed the development of lupus-associated pathogenic autoantibodies; we found that LBme suppressed the development of all lupus-associated autoantibodies tested over a 9-month (40 week) trial (anti-dsDNA, anti-Histone and ANA lupus autoantibodies, Figure 4). LBme was thus observed to prevent the development of pathologic autonomous B cell clones in genetically induced murine lupus as long as dosing was maintained.

**Figure 5:**
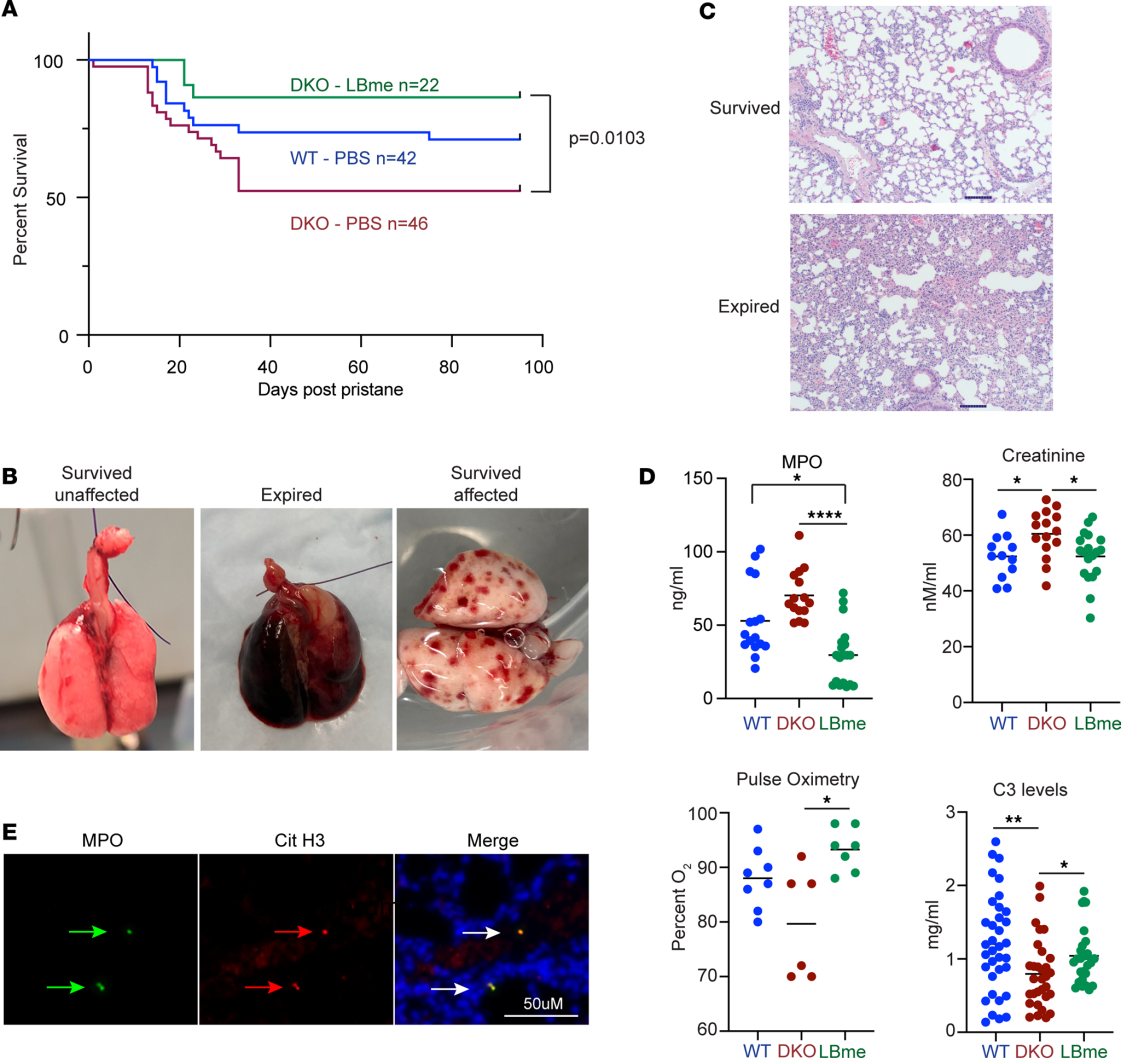
Survival and serum biomarkers of DKO mice following pristane challenged. (**A**) Following pristane challenge at 40 weeks, approximately 50% of vehicle-treated DKO mice died, whereas ≈85% of the DKO mice treated with LBme survived (*P* = 0.0103, Mantel-Cox). (**B**) Gross and (**C**) microscopic appearance of the lungs of surviving and dead animals. The lungs of the surviving animals could be classified in two groups — those that were grossly normal and those exhibiting fibrotic scarring indicative of earlier hemorrhagic events. H&E examination of pulmonary tissue from deceased mice shows alveolar wall thickening and inflammation. Scale bar: 200 μM. (**D**) Serum MPO, creatinine, and C3 levels at 40 weeks, and pulse oximetry levels in the pristane-treated lupus mice at 44 weeks (4 weeks following pristane treatment). *P < 0.05, **P < 0.01, ****P < 0.0001, ANOVA Kruskal-Wallis test. (**E**) Immunofluorescence from a lung section of an untreated mouse removed from the study due to severe DAH showing deposition of MPO (green arrows, left) and citrullinated histone H3 (Cit-H3) (red arrows, middle) in the alveolar walls and DAPI-stained nuclei in blue. Scale bar: 50 μM. Mice that died from DAH all revealed similar staining.

At 40 weeks, we accelerated the disease phenotype with pristane, a chemical inflammatory stimulant, and followed animal survival over the next 100 days, discovering that untreated DKO mice with elevated anti-dsDNA Ab levels were susceptible to increased mortality by 52 weeks (Figure 4, comparing anti-dsDNA Ab titers in surviving mice with at 40 and 52 weeks, red circle) and that ANA titers in the surviving vehicle-treated DKO mice at 52 weeks remained elevated. Within 100 days of pristane stimulation, ≈50% of the vehicle-treated DKO mice died from pulmonary hemorrhage in comparison with ≈15% of LBme-treated mice (*P* = 0.0103, log-rank, Mantel-Cox survival test, Figure 5A) and that ≈ 25% of vehicle-treated WT controls also died following pristane treatment. Pulmonary hemorrhage was evident by gross examination in all died mice (Figure 5B). Lipogranulomas in the adipose tissue and pulmonary scaring in the lungs was noted in surviving animals from all three cohorts, and thickened and inflamed alveolar walls were noted microscopically (Figure 5C), and anti-MPO and anti-Cit H3 colocalized in the alveolar walls, providing some evidence for the deposition of NETs in the lungs (Figure 5E). At 40 weeks, plasma biomarkers, MPO and creatinine, were significantly elevated in untreated DKO mice. Hypocomplementemia C3, a strong predictor of end-stage kidney disease (39), was also noted in vehicle-treated DKO mice in comparison with LBme-treated siblings. Oxygen saturation 4 weeks after pristane treatment was significantly decreased in vehicle-treated DKO mice in comparison to their LBme-treated siblings (Figure 5D).

In summary, the combined anatomic, histologic, and biomarker data suggested that pristane exacerbation of the lupus phenotype in DKO mice led to NETosis, acute respiratory compromise due to alveolar hemorrhage, renal damage, and death, the sequela of which could be prevented by LBme, our enzyme biologic with dual DNASE1 and DNASE1L3 activity.

**Figure 6:**
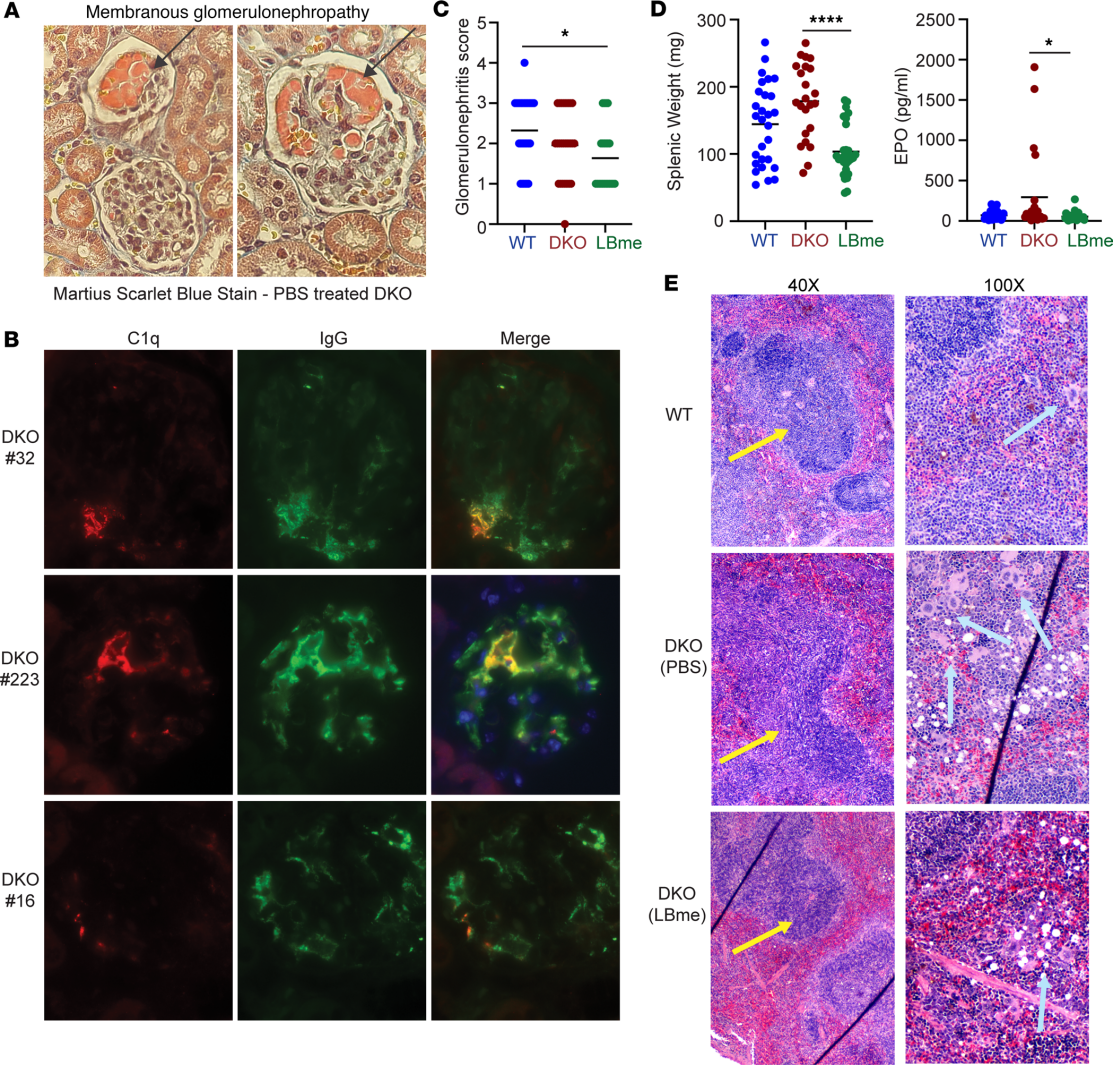
Membranous glomerulonephropathy and immune complex deposition in glomeruli of vehicle-treated DKO mice. (**A**) Histologic examination of the kidneys revealed the presence of membranous glomerulopathy and fibrin deposition (indicated by arrows) in some untreated DKO mice (Martius Scarlet Blue stain, original magnification, ×40). These findings were not present in the LBme-treated DKO cohort. (**B**) Examination of effected kidneys in untreated DKO mice revealed evidence of immunocomplex deposition via immunofluorescence staining with C1q (red) and IgG (green); merged images with DAPI-stained nuclei are also shown. Original magnification, ×100. (**C**) Glomerulonephritis assessed in a blinded fashion by a board-certified nephropathologist revealed a lower glomerulonephritis score in LBme-treated DKO mice than in WT controls, but there were no significant differences in the treated and untreated DKO mice. (**D**) Splenomegaly was significantly present in vehicle-treated DKO mice in comparison to their LBme-treated siblings, as were increased erythropoietin (EPO) levels at 52 weeks. (**E**) Histologic examination of the spleens revealed white pulp expansion due to coalescence of lymphoid follicles in vehicle-treated DKO mice (yellow arrows). Vehicle-treated DKO mice also exhibited robust extramedullary hematopoiesis in comparison to LBme-treated siblings and WT controls (cyan arrows). *P < 0.05, ****P < 0.0001, ANOVA Kruskal-Wallis test.

At 52 weeks all animals were sacrificed, and their organs were examined to assess the presence of tissue damage. Similar to DNASE1L3-deficient mice in the B6 background, we found that DKO mice did not develop significant glomerulonephritis compared with WT controls. However, membranous glomerulopathy was noted in some DKO mice that was not present in their LBme-treated siblings, some untreated DKO mice exhibited C1q colocalization with IgG in the glomeruli, and LBme-treated DKO mice showed a lower glomerulonephritis score than that of WT controls (Figure 6, A–C). Splenomegaly, however, was significantly present in vehicle-treated DKO mice in comparison to their LBme-treated siblings, as were elevations in erythropoietin (Figure 6D). Histologic examination of the spleens demonstrated coalescence of germinal centers and expansion of the white pulp in the vehicle-treated DKO mice in comparison with their LBme-treated siblings (Figure 6E, yellow arrows) as well as increased extramedullary hematopoiesis, as determined by the presence of megakaryocytes within the red pulp (Figure 6E, cyan arrows). In summary, in comparison with LBme-treated DKO mice, vehicle-treated DKO mice exhibited histologic evidence of chronic immune stimulation, as demonstrated by expansion of white pulp and splenomegaly, and increased hypoxia, as demonstrated by increased EPO levels and robust splenic extramedullary hematopoiesis. The constellation of findings suggested that the enzyme biologic ameliorated the immune stimulation, splenomegaly, and hypoxia in the model.

### LBme rescues mice from death in the pristane-induced lupus model.

**Figure 7:**
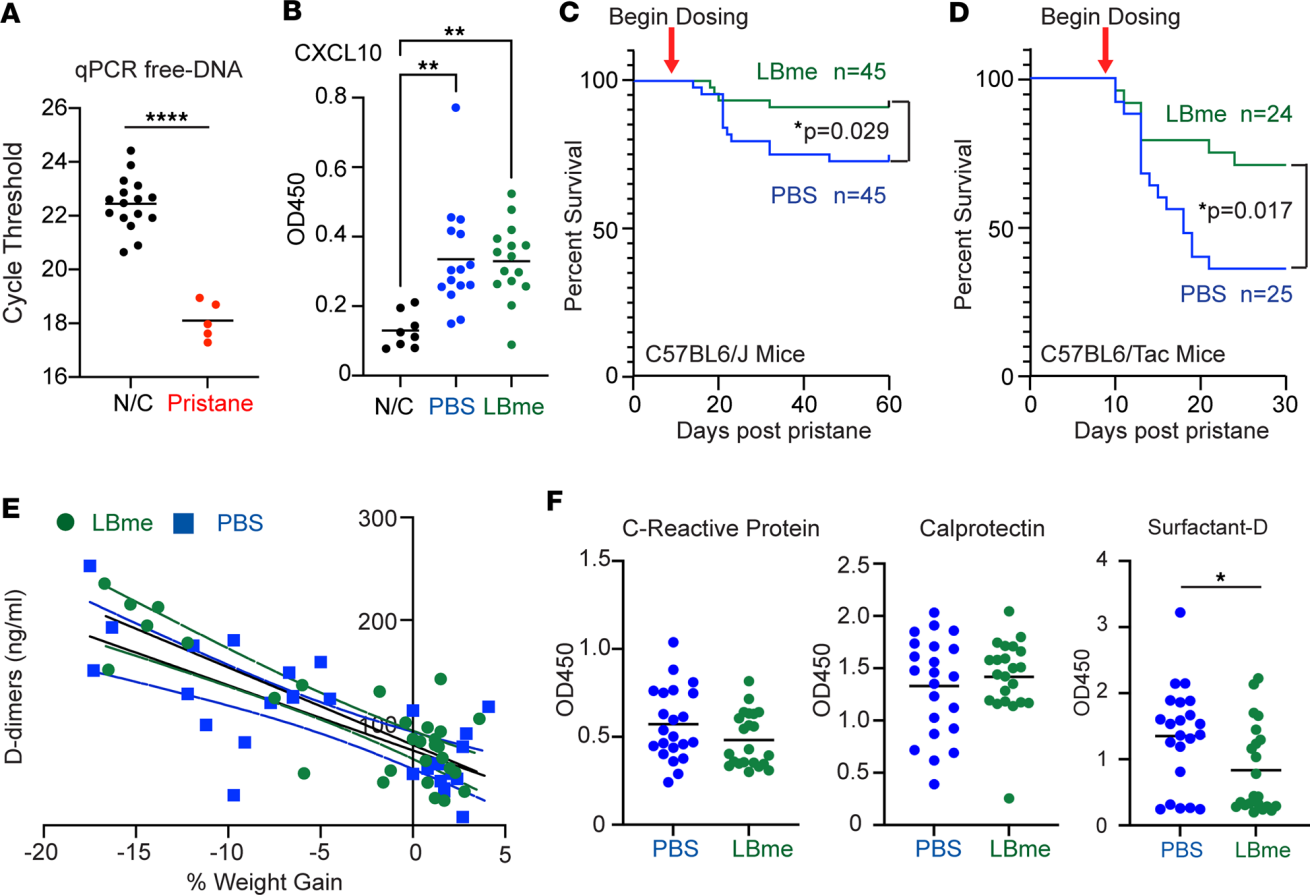
Efficacy of LBme in diffuse alveolar hemorrhage in 2 strains of C57BL/6 mice. (**A**) WT mice have higher amounts of plasma cfDNA 14 days after pristane injection than untreated negative control (N/C) mice when analyzed by qPCR. ****P < 0.0001, Student’s 2-tailed unpaired *t* test. (**B**) Ten days after pristane injection, before the dosing strategy began, both cohorts of mice show elevated levels of CXCL10 compared with N/C mice that did not receive pristane. **P < 0.01, ordinary 1-way ANOVA followed by Šidák’s multiple comparison test. (**C**) C57BL/6J mice (The Jackson Laboratory) were dosed i.p. with 500 μL pristane on day 0 and weekly with either PBS or LBme (1 mg/kg) following the first death of an animal after pristane challenge (on day 10). The survival rate of dosed and vehicle-treated animals was 95% and 70%, respectively (*P* = 0.029, Mantel-Cox). (**D**) The identical study as in **C** was performed in C57BL/6 mice (Taconic Biosciences), with dosing beginning on day 9 after pristane, yielding a survival rate of 70% and 35%, for dosed and vehicle-treated animals, respectively (*P* = 0.017, Mantel-Cox). (**E**) D-dimers measured in C57BL/6J mice at 14 days inversely correlated with weight gain in the vehicle- (slope = –6.35, *r*^2^ = 0.55, *F* = 33.01, *P* < 0.0001) and LBme-treated (slope = –7.54, *r*^2^ = 0.69, *F* = 63.84, *P* < 0.0001) cohorts. (**F**) C-reactive protein and calprotectin were equivalent in the treated and untreated cohorts, but surfactant-D levels were significantly higher in vehicle-treated C57BL/6 mice at 14 days after pristane challenge, demonstrating that although acute-phase reaction and NETosis was equivalent in treated and untreated cohorts, LBme reduced alveolar damage in the treated mice. *P < 0.05, Student’s 2-tailed unpaired *t* test.

The observation that the survival of LBme-treated DKO mice trended higher than that of vehicle-treated WT mice after challenge with pristane, and that even WT mice can show an increase in cfDNA after pristane treatment (Figure 7A), suggested that the enzyme biologic could provide a survival advantage to WT mice in a nongenetic murine lupus model, in which pristane was dosed to simulate the life-threatening complication of diffuse alveolar hemorrhage (DAH). To examine this hypothesis, we challenged two immunologically distinct C57BL/6 strains — one from Taconic Biosciences and the second from The Jackson Laboratory — with pristane. C57BL/6 mice from The Jackson Laboratory harbor an *Nlrp12* mutation that renders them less susceptible to neutrophil recruitment after an inflammatory stimulus than C57BL/6 mice from Taconic Biosciences (40). In this test, we began dosing with LBme, or vehicle control, after the first animal died following pristane stimulation to simulate a therapeutic dosing strategy in which treatment commences following the onset of symptoms. Mice were found to have significantly elevated levels of the C-X-C motif chemokine ligand 10 (CXCL10) at day 10, before dosing began, compared with negative control mice that did not receive pristane (Figure 7B). We found that LBme rescued mice from death in both strains, with the vehicle-treated mice from The Jackson Laboratory exhibiting ≈30% mortality compared with ≈10% in the LBme-treated cohort, whereas the vehicle-treated Taconic Biosciences mice exhibited ≈65% mortality compared with 30% mortality in the LBme-treated siblings (*P* = 0.029 and 0.017, respectively, Mantel-Cox) (Figure 7, C and D). Analysis of plasma biomarkers in treated and untreated animals 14 days after exposure to pristane revealed elevation of D-dimers in the animals, which correlated with weight loss in both treated and untreated animals (slope of –6.35 and –7.45, respectively, *P* < 0.0001) (Figure 7E). The findings are consistent with the diagnosis of disseminated intravascular coagulation (DIC), a bleeding diathesis characterized by rapid depletion of clotting factors due to extensive intravascular coagulation. Analysis of additional plasma biomarkers at 14 days revealed equivalent elevations in c-reactive protein and calprotectin, an abundant cytosolic protein in neutrophils, in both LBme- and vehicle-treated animals, with surfactant protein-D significantly elevated in the vehicle-treated mice compared with LBme-treated siblings (Figure 7F). These observations demonstrated that the acute phase response and disgorgement of neutrophilic cytosolic contents were equivalent in treated and untreated mice, but alveolar tissue damage resulting in leakage of intraalveolar surfactant was more pronounced in the untreated cohort. In summary, administering LBme to WT mice following the onset of respiratory distress and an initial fatality in the pristane model rescued animals from fatal pulmonary hemorrhage and death due to lung injury and a DIC-related coagulopathy.

### Additional characterization of the pristane lupus model.

**Figure 8:**
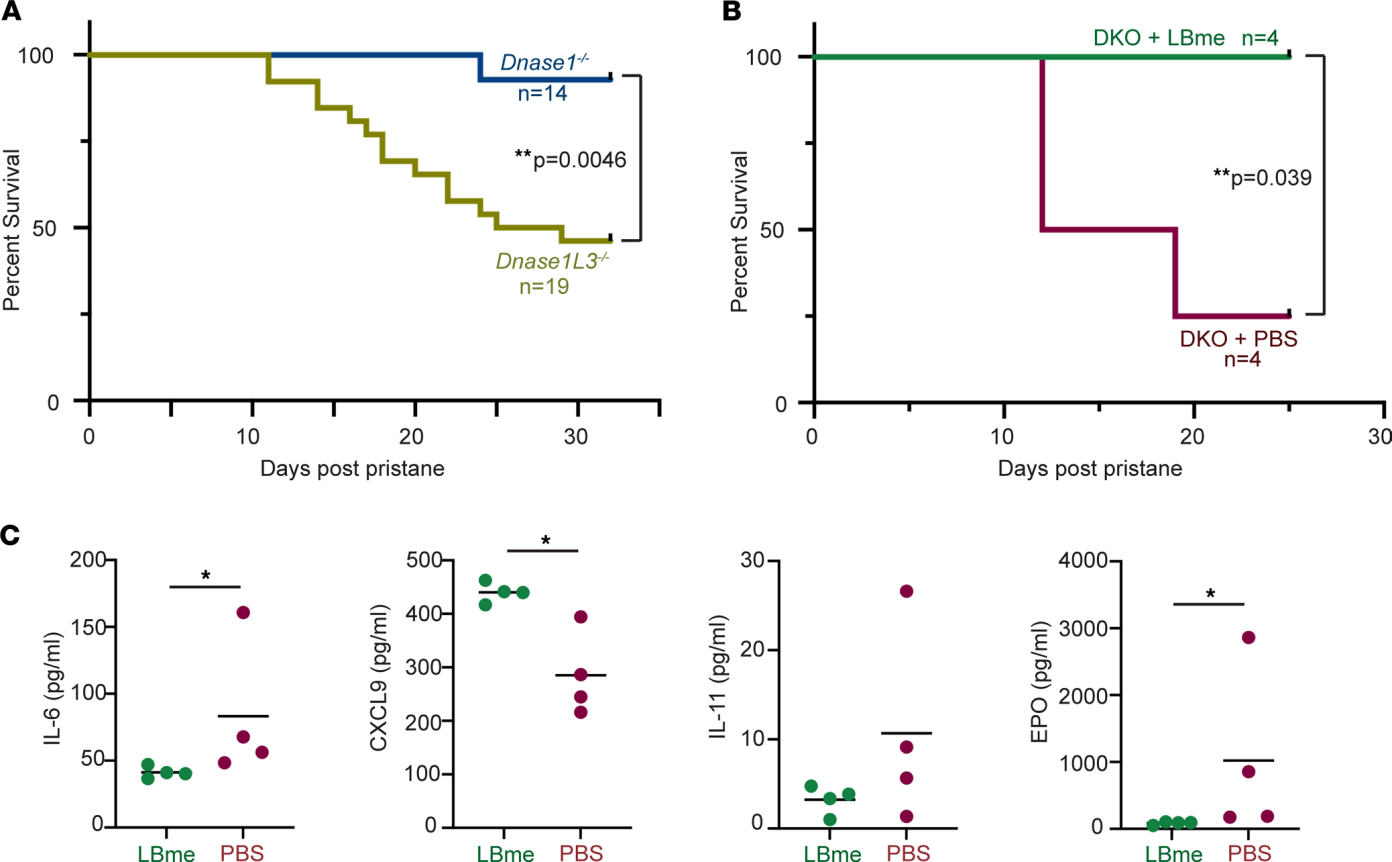
Additional characterization of the pristane-induced lupus model and the mitigating effects of LBme. (**A**) Survival comparison of *Dnase1^–/–^* and *Dnase1L3^–/–^* mice on C57BL/6J backgrounds following pristane challenge revealed increased mortality in *Dnase1L3^–/–^* mice compared with *Dnase1^–/–^* counterparts (50% vs. less than 10%, respectively, *P* = 0.0046, Mantel-Cox), illustrating the effect of functional loss of DNASE1L3 activity on the acute autoinflammatory phenotype. (**B**) The mortality induced by pristane challenge in DKO mice could be prevented with weekly 1 mg/kg s.c. doses of LBme (100% survival in dosed vs. 25% survival in vehicle-treated DKO mice, *P* = 0.039, Mantel-Cox). (**C**) Plasma biomarkers in the dosed and undosed DKO mice 10 days after pristane revealed higher levels of IL-6 and erythropoietin (EPO) in the undosed animals and lower levels of CXCL-9. IL-11 trended higher without significance in this limited (*n* = 4) study. *P < 0.05, 2-tailed Student’s unpaired *t* test.

To understand the relative contributions of DNASE1 and DNASE1L3 deficiency to the evolution of the coagulopathy and the acute complication of DAH, we challenged *Dnase1^–/–^* and *Dnase1L3^–/–^* mice generated on a C57BL/6 background with pristane. In following their survival over the next 40 days, we found that *Dnase1L3^–/–^* mice exhibited significantly greater mortality than *Dnase1^–/–^* mice following pristane stimulation (greater than 50% vs. less than 10% mortality, respectively, *P* = 0.0046, Mantel-Cox) (Figure 8A). To further characterize the efficacy of enzyme replacement, pristane-exposed mice were treated at day 0 and day 7 either with 1 mg/Kg LBme or with vehicle, and blood samples were collected at day 10. In this study, we prevented death in 100% of DKO mice treated with LBme, in contrast to vehicle-treated DKO mice, which exhibited 75% mortality (*P* = 0.039, Mantel-Cox) (Figure 8B). Plasma biomarkers in the animals revealed elevations in IL-6 and erythropoietin, and reductions in CXCL-9, in the vehicle-treated DKO animals, while IL-11 trended higher in the vehicle-treated cohorts without reaching significance (Figure 8C). These studies revealed that the effects of DNASE1L3 deficiency dominate the clinical phenotype induced by autoinflammation.

### Efficacy of the human isoform of LBme (1833) in plasma from patients with SLE.

**Figure 9:**
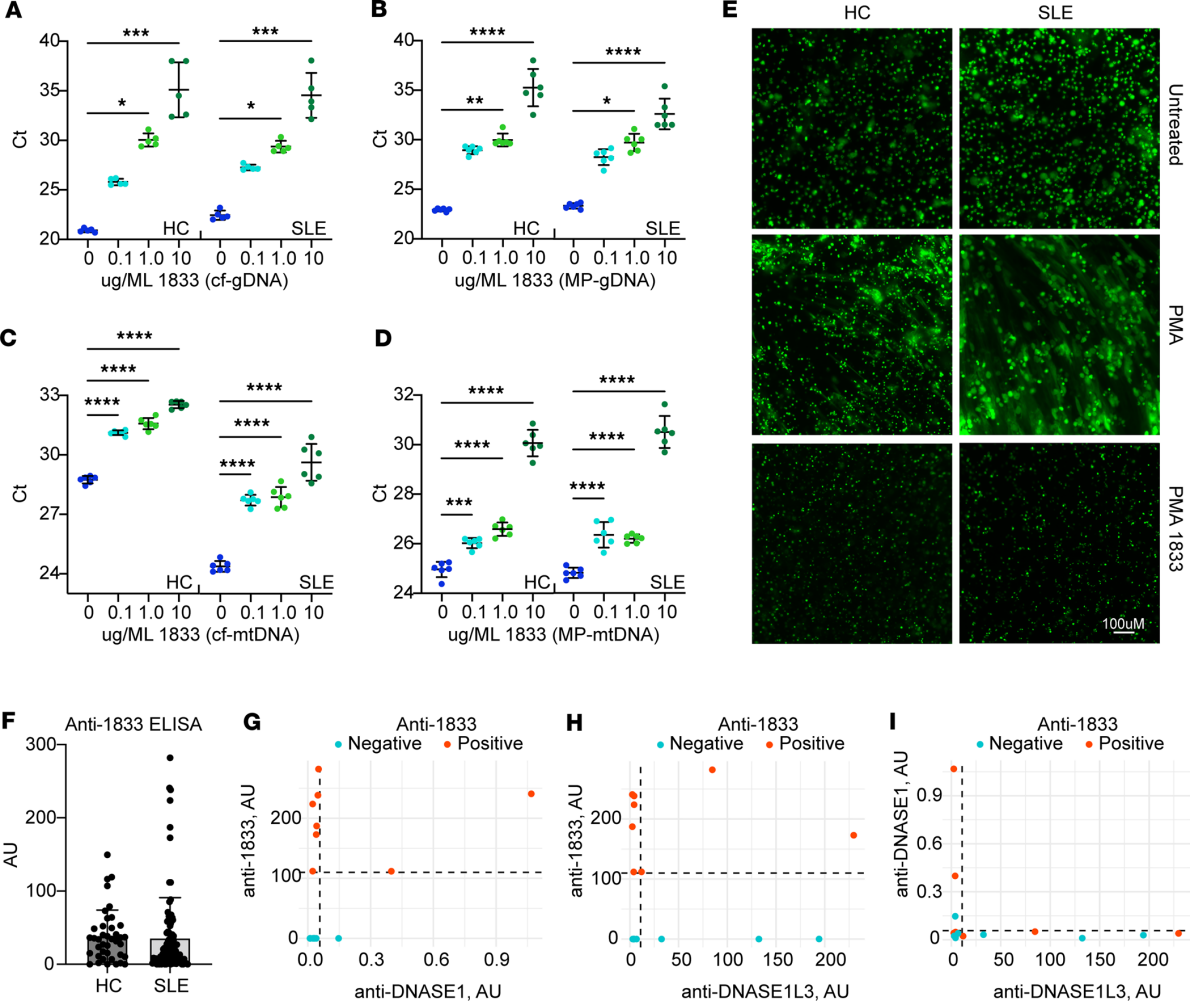
Efficacy of 1833 in the plasma of HCs and patients with SLE. Plasma, or the MP fraction of plasma, from a patient with SLE with a known titer of anti-dsDNA Ab and a HC was evaluated by qPCR (with technical replicates of *n* = 5–6) for the amount of (**A**) cf-gDNA, (**B**) MP-gDNA, (**C**) cf-mtDNA, and (**D**) MP-mtDNA remaining after an incubation with 0, 0.1, 1.0, or 10 μg/ML 1833 for 10 minutes at 37°C. Similar dose-response results were observed from 3 HC and 4 SLE patient samples evaluated. Statistics were determined using an ordinary 1-way ANOVA followed by Šidák’s multiple comparison test. (**E**) Images of Sytox Green fluorescence of PMA-stimulated leukocytes isolated from a HC and a SLE patient after an incubation with 50 nM 1833 for 10 minutes at 37°C demonstrating the efficient digestion of NETs by 1833. Representative images from an experiment performed twice with similar results from 3 HC and 4 SLE patient samples. Scale bar: 100 μM. (**F**) Abs against 1833 in HCs (*n* = 40) and SLE patients (*n* = 99) showing the mean plus 2 SD error bar. Anti-1833 Abs present in 8% (8 of 99) of SLE patients compared with 10% (4 of 40) of HCs. To confirm that 1833 autoantibodies present in the SLE plasma were not anti-DNASE1 or anti-DNASE1L3 autoantibodies, we exposed the plasma of the 8 SLE patients reactive to 1833 (*n* = 8, red dots) to human DNASE1 and DNASE1L3, comparing this reactivity to a similar number of randomly selected SLE patients negative for anti-1833 Abs (*n* = 8, blue dots). We found no significant correlation between (**G**) anti-1833 and anti-DNASE1 (*r* = 0.333, *P* = 0.226), (**H**) anti-1833 and anti-DNASE1L3 (*r* = –0.059, *P* = 0.8345), or (**I**) anti-DNASE1 and anti-DNASE1L3 reactivity (*r* = –0.230, *P* = 0.409). Correlations were tested using Pearson’s *r* test. Dashed lines show the cutoff for autoantibody positivity. Cutoffs for each autoantibody were determined as the mean plus 2 SD of the autoantibody titers on HCs (data not shown).

To demonstrate and characterize the efficiency of our biologic in patients with lupus, we began by quantifying the degradation of various types of cf and MP DNA in the plasma of 4 patients with SLE and 3 HCs. In a dose-response analysis, at concentrations of 0.1, 1.0, or 10 μg/mL, we were able to show, using qPCR, that the human isoform of LBme, called 1833, was able to efficiently degrade cf-gDNA, MP-gDNA, cf-mtDNA, and MP-mtDNA in both samples from HCs and patients with SLE (Figure 9, A–D, respectively, Ct results from one of the HCs and one of the patients with SLE with a known titer of anti-dsDNA Abs). We were also able to show that both 1833 and LBme could degrade cfDNA when added directly to whole blood, demonstrating that there should be no inhibition of the biologics in the circulation after dosing (Supplemental Figures 6 and 7). Next, we isolated leukocytes from HCs and patients with SLE and stimulated NET formation with PMA, demonstrating more robust NETosis in the SLE leukocytes, a finding which concurs with prior studies (30–32) (Figure 9D). Incubating these samples with 1833 at a final concentration of 50 nM for 10 minutes demonstrated the efficient degradation of the PMA-induced NETs derived from leukocytes in both HCs and patients with SLE (Figure 9E).

Finally, to confirm that 1833 would not be neutralized by autoantibodies in patients with SLE, we determined whether patients with SLE have Abs against 1833 as well as their relationship with anti-DNASE1 and anti-DNASE1L3 autoantibodies. Using a cohort of 99 patients with SLE and 40 HCs, we found no significant difference in their reactivity to 1833. Anti-1833 Abs were positive in 8% (8 of 99) of patients with SLE compared with 10% (4 of 40) in HCs (Figure 9F). Importantly, this finding contrasts with the prevalence of Abs against DNASE1 and DNASE1L3 (64% and 30%, respectively) (8, 22), indicating that autoantibodies against these DNASES are not cross-reactive with 1833. Indeed, only 2 of the 8 patients with SLE who tested positive for anti-1833 Abs were also positive for Abs against human DNASE1 (*r* = 0.333, *P* = 0.226), and 2 different patients tested positive for anti-DNASE1L3 Abs (*r* = –0.059, *P* = 0.8345) (Figure 9, G and H, respectively), further demonstrating that these are not the same autoantibodies. Similarly, Abs against DNASE1 did not correlate with anti-DNASE1L3 Abs in patients positive for anti-1833 Abs (*r* = –0.230, *P* = 0.409) (Figure 9I).

## Discussion

Loss of tolerance to self-DNA is a central defining feature of SLE, and class-switched autoantibodies against dsDNA are among its most reliable diagnostic serologic markers. Decades ago, the activity of plasma enzymes digesting DNA in patients with lupus was found to be low, and pathogenic variants of DNASE1 and DNASE1L3 have been identified in patients with lupus nephritis, while other studies have shown that the activity of DNASE1 is low in patients with sporadic lupus nephritis (41–43). Additionally, autoantibody-mediated impairment of DNASE1L3 activity has recently been identified as a common nongenetic mechanism facilitating the development of anti-dsDNA Abs in patients with severe sporadic SLE (8, 20). These combined observations led to the hypothesis that the anti-DNA response characterizing lupus may result from the inefficient clearance of extracellular DNA, whether in the form of cfDNA in blood, genomic self-DNA and/or chromatin in apoptotic MPs, or chromatin-associated DNA extruded by neutrophils during the process of NETosis. Unfortunately, experiments using a recombinant human DNASE1 to test this hypothesis in both humans and mice with lupus failed to show efficacy, discouraging further study. In retrospect, these studies were compromised both by the low-to-absent bioavailability of recombinant DNASE1 used and by the lack of DNASE1L3 activity required to degrade MP-DNA and/or NETs, both of which have recently been posited to be important antigenic drivers of lupus autoimmunity (26, 44, 45).

We sought to re-examine the importance of DNA clearance in the pathogenesis of lupus by engineering a long-acting, bioavailable, glycopolished enzyme biologic with dual DNASE1/DNASE1L3 activity for use in murine models of lupus. To do so, we engineered glycosylation consensus sequences onto the predicted surface of DNASE1 and DNASE1L3 to inhibit actin binding (in the case of DNASE1) and to extend the biologic’s half-life in vivo. We also attempted to interconvert the enzymatic activities by using directed amino acid substitutions near the enzymatic active sites followed by successive screening of DNASE1L3 activity in DNASE1 isoforms and vice versa. Our efforts yielded several clones containing positively charged amino acids near the active site of DNASE1 that successfully conferred DNASE1L3 activity onto the DNASE1 protein backbone (e.g., the combination of Q31R and N96K in human DNASE1, construct 1833). We were able to engineer glycosylation consensus sequences that increased the glycosylation repertoire of several isoforms, as illustrated by an increase in mobility shift on Coomassie-stained gels (Figure 2C). The V88N glycosylation was noted to prevent actin inhibition in construct LBme, and several others were noted to increase in vivo half-life (e.g., G262N in LBme).

Following in vivo pharmacodynamic testing to establish dose range and frequency using DKO mice lacking both DNASE1 and DNASE1L3, we tested the efficacy of the lead murine biologic isoform, LBme, on the development of autoimmunity and loss of tolerance to self-DNA in DKO mice. Whereas vehicle-treated DKO mice had elevated anti-dsDNA, anti-ssDNA, and antinuclear autoantibodies by 8 weeks of age, dosing DKO mice s.c. with 1 mg/kg LBme completely prevented the elevation of all autoantibodies for 40 weeks. At 40 weeks we accelerated the disease phenotype with pristane, which introduces cellular apoptotic debris and NETotic chromatin into the circulation (46, 47), resulting in a lethal complication of lupus called DAH. We found that 50% of the vehicle-treated DKO mice receiving pristane died from DIC and pulmonary hemorrhage within the next 12 weeks, compared with only 15% of LBme-treated DKO mice. At 52 weeks, histologic examination of the surviving animals revealed increased organ damage in the spleens and lungs of vehicle-treated DKO mice compared with LBme-treated animals, and glomerulonephritis was significantly reduced in LBme-treated DKO mice relative to vehicle-treated WT controls. Finally, plasma biomarkers revealed increased plasma erythropoietin and hypocomplementemic C3 levels in the vehicle-treated DKO mice, suggesting that LBme prevented hypoxia and ameliorated immune complex deposition in the DKO mice. The constellation of findings supports the notion that DNASE1 and DNASE1L3 activity is a protective mechanism for the development of autoimmunity in lupus.

DAH is an acute and life-threatening medical emergency. It has been recently recognized as a primary mechanism of mortality in severe COVID-19 infections, but it may also occur as a complication in autoimmune disorders (such as SLE, ANCA vasculitis, or antiphospholipid syndrome); following transplantation of solid organs or hematopoietic stem cells; as the sequela of bacterial or viral sepsis; and in trauma patients with severe burns. There is no specific therapy for DAH, and supportive care measures attempting to stabilize hemodynamic instabilities, correct coagulopathies, and provide ventilatory support are often unsuccessful, leading to a high acute mortality rate of 46% from all causes (48).

The strong efficacy of LBme in the genetic murine lupus model accelerated with pristane suggested that a therapeutic benefit may be present in nongenetic lupus models. To test this hypothesis, we tested the efficacy of LBme in pristane-stimulated strains of C57BL/6 WT mice and waited until the first animal died before beginning dosing to simulate a therapeutic dosing protocol. Unlike the C57BL6/N mice from Taconic Biosciences, the C57BL/6J mice from The Jackson Laboratory are known to harbor a point mutation in *Nlrp12* that reduces neutrophil recruitment during inflammation (40), providing a mechanistic understanding of the increased mortality observed in Taconic Biosciences mouse models of pristane-induced lupus, although both strains were be rescued from mortality by LBme. The mortality in the pristane model appears to result from a combination of alveolar injury and coagulopathy induced by DIC, closely paralleling the pathogenesis of lupus-induced and other immune-mediated DAH in humans. The survival advantage conferred by LBme treatment was robust, further strengthening claims that enhancing DNA degradation is likely to carry significant therapeutic benefit in autoinflammatory disease.

Our final murine studies examined the relative contributions of DNASE1 and DNASE1L3 to mortality present in the pristane-induced lupus model, conclusively demonstrating that DNASE1L3 activity drives this phenotype. Following pristane stimulation, *Dnase1L3^–/–^* mice exhibited greater than 50% mortality, as opposed to less than 10% mortality in the *Dnase1^–/–^* mice. The pathogenesis of DAH in C57BL6 mice after pristane injection has been shown to be associated with NETosis (47), a process whereby neutrophils release cytotoxic protein bound to chromatin in the form of NETs. Our observations illustrate the dramatic contribution of chromatin to disease phenotype in inflammatory disorders and support the notion that LBme is a dual-acting biologic capable of digesting chromatin in vivo. They also provide further support for the hypothesis that reduced DNASE1L3 activity is a primary driver of the cfDNA abnormalities and disease phenotype in autoimmune disorders and, indeed, that its absence may provide the antigenic trigger responsible for the loss of tolerance to self-DNA observed in these disorders.

A limitation in our study is the known lack of glomerulonephritis in DNASE1L3-knockout mice on a C57BL/6 background, which did not allow us to assess the effects of the therapeutic on renal nephritis. This limitation may have been further exacerbated by using pristane to accelerate mortality, which may have skewed death in animals with more severe renal disease. Therefore, although there was some indication that the therapeutic ameliorated renal damage, additional studies are necessary to investigate the efficacy of LBme in lupus nephritis murine models.

In conclusion, our studies suggest that enhancing the plasma activity of DNASE1 and DNASE1L3 with our bioavailable enzyme biologic is likely to carry significant therapeutic benefit in patients with autoimmune disease associated with impaired DNASE1L3 activity. This population not only includes the ultra-rare patients with DNASE1L3 variants described in the Arabic (9), Turkish (10), and Italian (11) population with SLE and/or a closely related autoimmune nephritic disorder, hypocomplementemic urticarial vasculitis syndrome, but also patients with SLE with a more common hypomorphic variant of DNASE1L3 — rs35677470 — resulting in an R206C substitution in DNASE1L3 that substantial impairs secretion into the plasma (19). This variant is present in 10.4% of the Turkish, 15.4% of the German, and 2.5% of the Mexican population, with a mean allele frequency of 0.052, 0.077, and 0.017, respectively (13) and is associated with an increased risk of anticentromeric-positive systemic sclerosis, rheumatoid arthritis, idiopathic inflammatory myopathies, and SLE (where it is often identified as a SNP in a neighboring gene, *PXK*) (15, 18, 49–51). Moreover, patients with inactivating autoantibodies against DNASE1L3 recently described in sporadic SLE broaden the pool of patients who may benefit from this approach (8, 20). To address this latter population, we demonstrated that the human isoform of LBme — 1833 — is not a significant target of Abs in patients with SLE and showed that 1833 effectively degraded cf and MP genomic and mitochondrial DNA in the plasma and whole blood of patients with SLE as well as NETs derived from their leukocytes.

Our combined studies validate 1833 as an enzyme therapeutic for patients with autoimmune disorders associated with DNASE1L3 deficiency. Framing the disease pathogenesis in this context, protein replacement carries an astounding FDA regulatory approval rate of 88%–91% in comparison with 19% for all other classes of drugs (52), which speaks to both the predictive power of the preclinical monogenic murine disease models and to the translational potential of enzyme biologics as safe and effective therapeutics for patients with autoimmune and autoinflammatory disorders associated with DNASE1L3 deficiency.

## Methods

### Sex as a biological variable.

The experimental results using both male and female mice were considered separately and then combined, as similar findings were observed for both sexes.

### Creation of a long-acting, hyperactive, bioavailable enzyme therapeutic with dual DNASE1 and DNASE1L3 activity.

Mouse *Dnase1* cDNA was amplified from a C57BL6/J cDNA library and cloned in-frame into the plasmid pFUSE-mIgG1-Fc1 (InvivoGen) to yield the parent DNASE1-Fc fusion protein, which consisted of 284 amino acids of the mouse DNASE1 fused by 7 amino acids to 222 amino acids of the CH2 and CH3 domains of mouse IgG heavy chain and hinge region. For the human version, cDNA for human DNASE1, codon optimized for CHO cell expression, was obtained from Integrated DNA Technologies and cloned in-frame into pFUSE-hIgG1-Fc1 (InvivoGen). Subsequent mutations were performed using QuikChange II XL Site Directed Mutagenesis (Agilent Technologies). All constructs were sequenced-verified before transfecting into CHO cells for protein production. Analysis of the purified protein is provided in the Supplemental Methods.

### DNASE1 and DNASE1L3 activity assays.

DNASE1 activity was determined by reacting 10 μL conditioned CHO cell media, 10 ng purified protein, or 5 μL plasma from a previously dosed mouse in a 20 μL solution containing 1 μg plasmid DNA, 100 mM Tris pH 7.5, 3 mM CaCl2, 3 mM MgCl_2_, and 50 mM NaCl for 5–10 minutes at 37°C and visualized on a 1% agarose gel. To analyze DNASE1L3 activity, nuclei were isolated from Wehi-3 cells (ATCC) using the Nuclei Isolation Kit (MilliporeSigma) and incubated in the same buffer in a final volume of 60 μL. After 30–45 minutes at 37°C, the DNA was extracted by adding 300 μL 7 M guanidine HCl, before being transferred to a Qiagen mini-prep spin column, washed, eluted, and run out on a 2.5% agarose gel. In some experiments, DNASE1 and DNASE1L3 activities were measured in mouse serum or urine without any buffer by adding plasmid DNA or purified Wehi-3 nuclei directly to serum.

### Quantitation of cfDNA from plasma or urine by qPCR.

To quantitate circulating cfDNA from mice, blood from a retro-orbital bleed in EDTA was centrifuged at 1,400*g* for 10 minutes. The top layer was separated from the Buffy coat, transferred to a new tube, and centrifuged at 14,000*g* for 10 minutes, and the supernatant was transferred to a new tube. For qPCR we used 1 μL platelet-free plasma or 1 μL urine in a 20 μL solution of 1x SsoAdvanced Universal Inhibitor-Tolerant SYBR Green Supermix (Bio-Rad) with the following DNA oligonucleotide sequences designed to hybridize to mouse retrotransposons: sense 5′ CCTCTAGTGAGTGGAACACAACTTCTGC 3′ and antisense, 5′ TGCAGGCAAGCTCTCTTCTTGC 3′. Ct values are displayed in Prism GraphPad, and statistical significance was calculated using a nonparametric Mann-Whitney *t* test.

### Dose-response digestion of HC and SLE patient cf-gDNA and cf-mtDNA using 1833.

Platelet-free plasma was isolated from freshly drawn whole blood into EDTA tubes by transferring the top layer of plasma into a new tube after 3 rounds of centrifugation: first, 500*g*; then, 2,000*g*; followed by a third round of centrifugation at 14,000*g*; each for 10 minutes at 4°C. The plasma was then supplemented with 20 mM each of CaCl_2_ and MgCl_2_ before adding 0, 0.1, 1.0, or 10 μg/ML 1833 and incubated for 10 minutes at 37°C before being placed on ice. For qPCR, 1 μL of the digested plasma was added directly into a 20 μL reaction using 1x SsoAdvanced Universal Inhibitor-Tolerant SYBR Green Supermix (Bio-Rad) with DNA oligonucleotide sequences designed to hybridize to either human LINE1 5′ UTR (5′ CGAGATCAAACTGCAAGGCG and 5′ CCGGCCGCTTTGTTTACCTA) or the human mitochondrial tRNA-Phe gene (5′ CTAAATAGCCCACACGTTCCC and 5′ AGAGCTCCCGTGAGTGGTTA). Ct values are displayed in Prism GraphPad, and statistical significance was determined using an ordinary 1-way ANOVA followed by Šidák’s multiple comparison test if the residuals passed normality testing (α ≤ 0.05). If the residuals failed normality testing, ANOVA comparison of means using a nonparametric Kruskal-Wallis independent test with Dunn’s post hoc analysis was employed.

### Dose-response digestion of HC and SLE patient MP-gDNA and MP-mtDNA by 1833.

The MP fraction of whole blood was isolated from 500 μL platelet-free plasma (described above) using a fourth round of centrifugation at 21,000*g* for 1 hour at 4°C. After carefully aspirating the plasma, the MP pellet was washed with 1 mL PBS followed by another round of centrifugation at 21,000*g* for 1 hour at 4°C. The final MP pellet was resuspended in 100 μL PBS, from which 10 μL was withdrawn and added to an equal volume of digestion buffer (200 mM Tris pH 7.5, 50 mM NaCl, 5 mM CaCl_2_ and 5 mM MgCl_2_) containing a final concentration of 1883 equal to 0, 0.1, 1.0, or 10 μg/ML 1833. The replicates were incubated at 37°C for 10 minutes, and the reaction was stopped on ice. From the reaction, 1 μL was withdrawn and used in a qPCR reaction for the analysis of MP-gDNA and MP-mtDNA as described above.

### Digestion of NETs from human leukocytes by 1833.

Leukocytes were isolated by adding 10 mL of ACK buffer (MilliporeSigma) to 1 mL whole blood from either patients with SLE or a HC for 5 minutes at room temperature to lyse RBCs, followed by centrifugation at 300*g* for 5 minutes. The leukocyte pellet was washed with PBS and then plated into a poly-lysine–coated optical bottom 96-well plate (Thermo Scientific) in RPMI without FBS and allowed to adhere for 30 minutes before adding PMA to a final concentration of 50 nM. After 2 hours at 37°C to induce NETs, 50 nM 1833 was added for an additional 10-minute incubation at 37°C, and then paraformaldehyde was added to 2% and the plate removed and fixed at 4°C. The next day, 5 μM cell impermeable Sytox Green (Thermo Scientific) was added, and NETs were imaged on a BZ-X Keyence fluorescence microscope with 488 nm laser light and emission collection at 449–552 nm.

### In vivo pharmacokinetics.

DKO mice were injected s.c. with 1 mg/kg of the biologic, and both DNASE1 and DNASE1L3 activity were measured from sera at 2, 6, and 11 days after injection from at least 4 biological replicates per biologic.

### Genetic model of murine lupus.

A detailed description for the creation of the DKO mice is provided in the Supplemental Methods. The experimental results for both sexes were considered separately and then combined, as similar findings were observed for both sexes. A total of 126 randomly assigned WT and DKO mice were injected s.c. weekly with either PBS or biologic at 1 mg/kg, starting at 2 weeks of age. Sera and plasma were collected via retro-orbital bleed from mice at 8, 14, 25, 40 and 52 weeks, and autoantibodies were measured by ELISA. At the 40-week bleed, all mice in the lupus study were i.p. injected with 500 μL pristane (MilliporeSigma). The surviving mice were euthanized at 52 weeks by cervical dislocation after isoflurane anesthesia. Kidneys, spleen, and lungs were removed, fixed in 10% neutral buffered formalin overnight, and paraffin-embedded for histological analysis. Spleens were weighed after overnight fixation before embedding. For immunofluorescence, unstained slides were deparaffinized and heated in a pressure cooker for 5 minutes in 10 mM sodium citrate pH 6.0 for antigen retrieval. After blocking with 5% BSA in PBS containing 0.5% Tween-20 (PBS-T), slides were incubated with primary Abs, washed with PBS-T, and followed with Alexa Fluor–conjugated secondary Abs ab150121, ab150120, ab150068, and ab96935 (Abcam). Primary Abs used included anti-MPO (AF3667) and ST6GAL1 (AF5924) (R&D Systems) and anti-histone H3 (citrulline R2+R8+R17; AB5103) and anti-C1q (AB155012; Abcam).

### ELISA.

Anti-dsDNA ELISAs were created using MaxiSorp 96 well plates (NUNC) pretreated with 0.01% poly-Lysine in PBS for 2 hours at room temperature or overnight at 4°C, washed with PBS-T and coated with Calf Thymus DNA (Sigma-Aldrich) at 1 μg/well in PBS overnight at 4°C. Plates were washed 3 times and blocked with 5% BSA, 1% normal goat serum PBS-T for 2 hours at room temperature or overnight at 4°C. After washing 3 times, plates were incubated with mouse sera or plasma at 1:300 dilution in 1% BSA PBS-T for 2 hours at room temperature or overnight at 4°C. Plates were washed 4 times with PBS-T and incubated with a 1:2,000 dilution of goat anti-mouse IgG HRP-conjugated secondary Ab (ab97023) or IgM (ab97230) (Abcam) for 1 hour, washed again 4 times, and incubated with 100 μL per well of 1-Step Ultra TMB (Thermo Fisher Scientific) until desired color developed. The reaction was terminated with 2 M sulfuric acid, and absorbance measured at OD = 450 nm on a Synergy Mx microplate reader (BioTek). The ELISA for analysis of anti-ssDNA autoantibodies was performed the same way, with the exception that the Calf Thymus DNA was first sonicated and then heat denatured at 98°C for 10 minutes and placed immediately on ice before plating out in poly-lysine 96-well plates at 4°C as above. Anti-histone ELISA was performed similarly by binding 10 μg/mL histone from calf thymus (Roche Diagnostics) in 100 mM sodium carbonate, pH 9.4. For the detection of ANAs, Wehi-3 cells (ATCC) were fixed with ice-cold methanol for 5 minutes, washed with PBS-T, and blocked with 5% BSA in PBS-T before incubating with a 1:300 dilution of mouse sera or plasma. For the 14-week samples, the secondary Ab was a goat anti-mouse Alexa Fluor conjugate that was visually evaluated on a scale from 1 to 4 for signal intensity under a fluorescence microscope. The 25-week samples were prepared similarly but the evaluation was performed by measuring the fluorescence signal in each well using NIH ImageJ software. The 40- and 52-week ANA samples used a goat anti-mouse HRP secondary Ab followed by 1-Step Ultra TMB as above. Commercial ELISA assays were used for the following analytes: C-X-C chemokine motif ligand 10 (ab100675), creatinine (ab65340), C-reactive protein (ab222511), EPO (ab270893), C3 (ab263884), calprotectin (ab263885), and surfactant-D (ab240683) (Abcam). Additional data for EPO, IL-6, CXCL9, and IL-11 were obtained from an EVE Technologies Mouse Cytokine/Chemokine 44-Plex Discovery Array. Abs against 1833 and recombinant human DNASE1 (SinoBiological, 13801-H08H ab275555) were detected by ELISA. Briefly, Nunc Maxisorp plates were coated with 200 ng target peptide. The plates were blocked for 1 hour with PBS-T with 3% nonfat milk. The sera and plasma were diluted 1:1,000 in PBS-T 1% nonfat milk and assayed in duplicate using antigen-conjugated plates and plates without antigen for background subtraction. HRP-conjugated goat anti-human IgG was used as a secondary Ab (diluted at 1:10,000 in PBS-T 1% nonfat milk). Anti-1833 Ab arbitrary units were calculated using a standard curve made of a serial diluted serum from a high-titer patient with SLE. The cutoff for anti-1833 positivity was defined as 2 SD above the mean of anti-1833 in HCs. Abs against DNASE1L3 were detected as previously described (11) as follows. Radiolabeled DNASE1L3 was immunoprecipitated using 2 μL serum in 300 μL NP-40 buffer (20 mM Tris/HCl, 150 mM NaCl, 1 mM EDTA, 1% Nonidet P40, pH 7.4) for 1 hour at 4°C. Protein A beads were added and incubated for an additional 30 minutes at 4°C. After 3 washes with vortexing in NP-40 lysis buffer, the beads were boiled in SDS sample buffer. Samples were separated by gel electrophoresis, and radiography was used to visualize the immunoprecipitated proteins. Densitometry was performed on all films, and the results were normalized to a high-titer anti-DNASEL13 serum. Ab positivity was determined as 2 SDs above the mean anti-DNASE1L3 Ab level in HC sera.

### Pristane-induced lupus model.

10- to 14-week-old WT and DKO mice were injected i.p. with 500 μL pristane and weighed every 2–3 days until a 5% loss in body weight was detected, and then they were weighed every day thereafter. For the preventative study, DKO mice were randomly divided into 2 cohorts and injected s.c. weekly with either 1 mg/kg biologic or PBS starting on the same day as pristane injection. In the therapeutic study, WT mice were randomly divided into 2 cohorts and injected weekly with s.c. doses of either 1 mg/kg enzyme biologic or vehicle (PBS) beginning after the first mouse died, which was 9 days after pristane exposure for C57BL/6 mice from Taconic Biosciences, and 10 days after pristane exposure for C57BL/6 mice from The Jackson Laboratory. Mice were euthanized and listed as nonsurvivors after the loss of greater than 25% body weight or if the mice exhibited a significantly hunched posture or lethargic and labored breathing was detected. All nonsurviving (euthanized) animals in the pristane trials exhibited visible pulmonary hemorrhage upon gross examination of the lungs and histologic evidence of alveolar damage upon microscopic examination of lung histology. Arterial oxygen saturation (SpO_2_) readings were obtained using a MouseOx Plus Pulse Oximeter (STARR Life Sciences) on mice anesthetized under isoflurane.

### Statistics.

GraphPad Prism 10 was used to statistically analyze all data. Comparisons between two groups were performed using a 2-tailed Student’s unpaired *t* test. Survival comparisons between treated and untreated mice in all animal models were determined using the log-ranked (Mantel-Cox) test. Linear regression was performed with a simple linear regression model using Pearson *r* correlations and *P* values to determine significance. The statistical significance of ELISA assays among 3 groups was determined using an ANOVA comparison of means using the nonparametric Kruskal-Wallis independent test at a significance level of α = 0.05. Statistical significance among 3 or more groups for Ct values after qPCR was determined using an ordinary 1-way ANOVA followed by Šidák’s multiple comparison test if the residuals passed normality testing (α ≤ 0.05). If the residuals failed normality testing, ANOVA comparison of means using a nonparametric Kruskal-Wallis independent test with Dunn’s post hoc analysis was employed. All statistical testing used a significance level of α ≤ 0.05, and *P* values of less than 0.05 were considered significant. Correlations were tested using Pearson’s *r* test.

### Study approval.

Animal procedures were approved by the Animal Care and Use Committee of Yale University (Animal Protocol no. 2022-11535) and complied with the US NIH *Guide for the Care and Use of Laboratory Animals* (National Academies Press, 2011). Human studies were approved by the Hopkins Lupus Cohort under IRB NA_00039294 and NA_00001566 and by the Yale IRB under the Yale Lupus and Connective Tissue Disease Biorepository (HIC no. 1602017276). All patients consented in writing before inclusion.

### Data availability.

All data are available in Supporting Data Value file. The sequences of the LBme (accession PP213480) and the human version, 1833 (accession PP213481), are available in GenBank.

## Author contributions

SK designed and conducted research, provided essential reagents, and edited the manuscript. DS designed and conducted the research studies, acquired and analyzed the data, and edited manuscript. AB, DK, and QL designed and conducted research, acquired and analyzed the data, and edited the manuscript. SZ, JPP, KDB, and KJY designed and conducted research studies, provided essential reagents, and edited manuscript. ERL, HK, SGL, SS, and TI designed and conducted research studies, acquired data, provided essential reagents and edited the manuscript. DWG, FK, and MP designed and conducted the research, acquired and analyzed the data, provided essential reagents, and edited the manuscript. PRS, EGB, FA, and DTB designed and conducted research studies, acquired and analyzed the data, provided essential reagents, designed and executed the figures, and wrote and edited the manuscript.

## Supplementary Material

Supplemental data

Supporting data values

## Figures and Tables

**Table 1:**
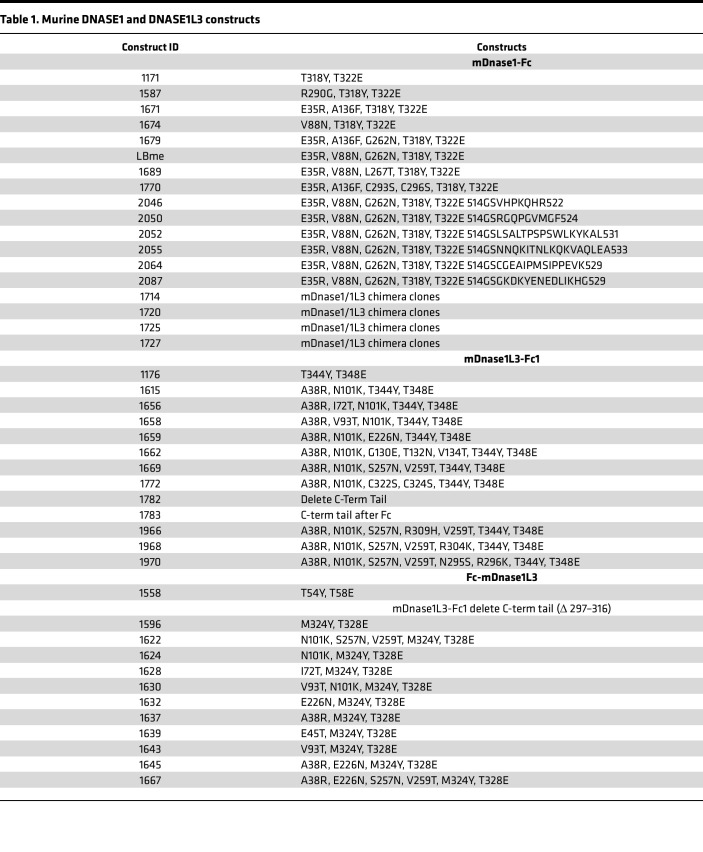
Murine DNASE1 and DNASE1L3 constructs

**Table 2:**
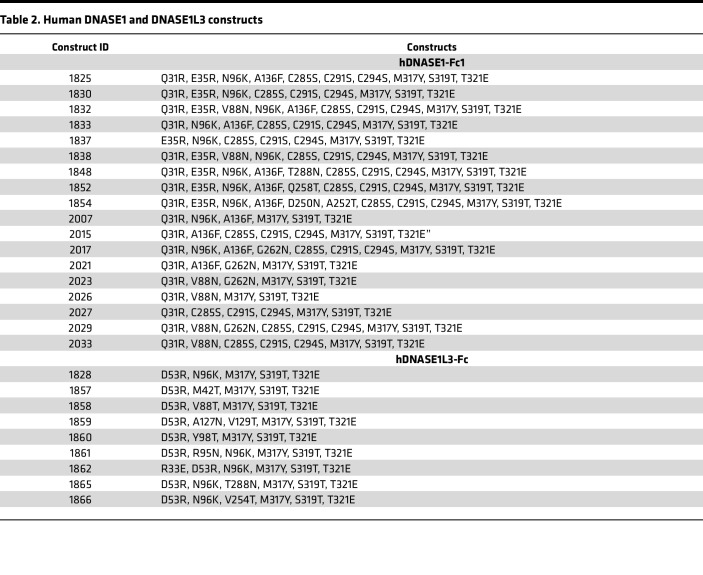
Human DNASE1 and DNASE1L3 constructs

## Version 1 06/18/2024

In-Press Preview

## Version 2 07/22/2024

Electronic publication
