# Supplementary material for: A dual-acting DNASE1/DNASE1L3 biologic prevents autoimmunity and death in genetic and induced lupus models
Source: JCI Insight. 2024 Jun 18;9(14):e177003. doi: 10.1172/jci.insight.177003 (PMC11383374; doi:10.1172/jci.insight.177003)
Supplement: Supplemental data [file jciinsight-9-177003-s125.pdf]

Supplemental Methods, Figures, and Figure Legends for:

A dual-acting DNASE1 biologic prevents autoimmunity and death in genetic and non-genetic lupus models.

Paul R. Stabach[1], Dominique Sims[1], Eduardo Gomez-Bañuelos[2], Sandra Zehentmeier[3], Kris Dammen-Brower[4], Andrew Bernhisel[1], Sophia Kujawski[5], Sam G. Lopez[1], Ethan R. Lester[1], Quan Le[1], Tayyaba Ishaq[1], Hana Kim[1], Shivani Srivastava[1], Deepika Kumar[1], Joao P. Pereira[3], Kevin J. Yarema[4], Fotios Koumpouras[5], Felipe Andrade[2], Demetrios T. Braddock[1]

[1]Department of Pathology, Yale University School of Medicine, New Haven Connecticut, 06510 USA
[2]Division of Rheumatology, The Johns Hopkins School of Medicine, Baltimore Maryland, 21224 USA
[3]Department of Immunobiology and Yale Stem Cell Center, Yale University School of Medicine, New Haven Connecticut, 06510 USA.
[4]Translational Tissue Engineering Center and the Department of Biomedical Engineering, The Johns Hopkins University, Baltimore Maryland, 21231 USA
[5]Department of Rheumatology, Yale University School of Medicine, New Haven Connecticut, 06510 USA.

*Address for Correspondence:
D.T. Braddock, M.D./Ph.D.
Department of Pathology
Yale University School of Medicine
310 Cedar Street
New Haven, CT 06510 USA
+1-203-737-1278
demetrios.braddock@yale.edu

Analytical HPLC to assess biologic purity: Protein sample (15μg in PBS) was dried at 25°C in SpeedVac for 20 minutes and reconstituted in 30 μL with 95% water 5% acetonitrile and 0.1% trifluoracetic acid. LC-PDA analysis was performed on a Waters H-Class UPLC system (Waters Technologies) utilizing a quaternary solvent system (Buffer A: 99.9% water, 0.1% trifluoracetic acid; Buffer B: 28.6% water, 71.4% acetonitrile, 0.075% trifluoracetic acid). Protein was profiled using an ACQUITY UPLC Protein BEH C4 Column 1.7 μm, 2.1 mm x 150 mm (40°C) and eluted at 0.4 mL/min with the following gradient: 28% buffer B at initial conditions, 100% B at 40 min; maintain 5 min, return to initial conditions at 50 minutes and maintained 20 minutes. The protein was detected in PDA detector with 220 nm channel.

Size exclusion - light scattering chromatography: The light scattering data were collected using a Superdex S-200, 10/30, HR Size Exclusion Chromatography column (GE Healthcare), connected to an HPLC system, *Alliance* 2965, (Waters Corporation) equipped with an autosampler. The elution from SEC was monitored by a photodiode array (PDA) UV/VIS detector (996 PDA, Waters Corporation), differential refractometer (OPTI-Lab, or OPTI-rEx Wyatt Corporation,), and static, multiangle laser light scattering detector (DAWN-EOS, Wyatt Corporation). The SEC-UV/LS/RI system was equilibrated in PBS at a flow rate of 0.5 ml/min. The weight average molecular mass, Mw, for the protein sample was tested at two concentrations of 0.59 mg/ml and 0.08 g/ml (1/20th dilution). Two software packages were used for data collection and analysis: the Millennium software (Waters Corporation) controlled the HPLC operation and data collection from the multi-wavelength UV/VIS detector, while the ASTRA software (Wyatt Corporation) collected data from the refractive index detector, the light scattering detectors, and recorded the UV trace at 280 nm, 295 nm, or 310 nm sent from the PDA detector; 295 and 310 nm were used for monitoring the elution of protein-DNA complexes, or for protein alone for when A_{280} >1.

Generation of *Dnase1/Dnase1L3*-double-deficient (DKO) mice. The *Dnase1L3* cKO mouse model was generated via CRISPR-Cas9 methods (1-3). Potential Cas9 target guide

(protospacer) sequences in introns 3 and 4 were screened using the online tool CRISPOR (http://crispor.tefor.net) (4), and candidates were selected. Templates for sgRNA synthesis were generated by PCR, and sgRNAs were transcribed in vitro and purified (Megashortscript, MegaClear; ThermoFisher). sgRNA/Cas9 RNPs were complexed and tested for activity by zygote electroporation, incubation of embryos to blastocyst stage, and genotype scoring of indel creation at the target sites. The sgRNAs that demonstrated the highest activity were selected for creating the floxed allele. Guide RNA (gRNA) sequences are as follows: 5' guide: TCCAGAGCAGGCTCAAGTGG and 3' guide ACCTGACACAAATACCTTGG. Accordingly, a 550 base long single-stranded DNA (lssDNA) recombination template incorporating the 5' and 3' loxP sites was synthesized (Integrated DNA Technologies). The injection mix of sgRNA/Cas9 RNP + lssDNA was microinjected into the pronuclei of C57Bl/6J zygotes (3). Embryos were transferred to the oviducts of pseudo pregnant CD-1 foster females using standard techniques (5). Genotype screening of tissue biopsies from founder pups was performed by PCR amplification and Sanger sequencing to verify the floxed allele. Germline transmission of the correctly targeted allele (i.e., both loxP sites *in cis*) was confirmed by breeding and sequence analysis. Subsequently, positive mice were mated with a Beta Actin CRE mouse to generate constitutive *Dnase1L3*-deficient mice. The *Dnase1*-deficient mouse strain, C57BL/6NDnase1tm1.1(KOMP)Vlcg/TcpMuncd, RRID:MMRRC_047412-UCD was obtained from the Mutant Mouse Resource and Research Center (MMRRC) at the University of California, Davis. *Dnase1*-deficient mice were mated with *Dnase1L3*-deficient mice to generate the *Dnase1/Dnase1L3*-double deficient mice, hereafter referred to as DKO mice.

Neutrophil Extracellular Trap (NET) digestion assay. Neutrophils were purified from mouse bone marrow as previously described (6). In brief, bone marrow was flushed from the mouse femur with a 26g needle filled using RPMI supplemented with 10% FBS and 2mM EDTA and

passed through a 70uM cell strainer and cells collected by centrifugation at 450 x g for 7min at 4°C. Cell pellet was resuspended in 20 ml of 0.2% NaCl for approximately 20 seconds followed by addition of 20 ml of 1.6% NaCl, followed by centrifugation at 450xg for 7min at 4°C. After washing cells again in RPMI, the cells were resuspended in 1mL PBS and layered on top of a Histopaque 1119/1077 density gradient, spun at 872 x g for 30 min without break and neutrophils were collected from the interface, washed with RPMI without supplement, plated out into 96 wells cell culture plate at 2.5 x 10(5) cells per well and incubated at 37°C in 5% CO2. NETs were induced by the addition of 50nM PMA with or without a serial dilution from 25-0.325 nM of either Roche DNASE1, LBme, or 1833. After 4 hours, 5uM cell impermeable DNA binding Sytox Green (ThermoFisher Scientific) was added and NETs were visualized on a BZ-X Keyence fluorescence microscope with 488nm laser light and emission collection at 449–552 nm. The level of Sytox Green fluorescence was quantitated on a Synergy Mx microplate reader (BioTek).

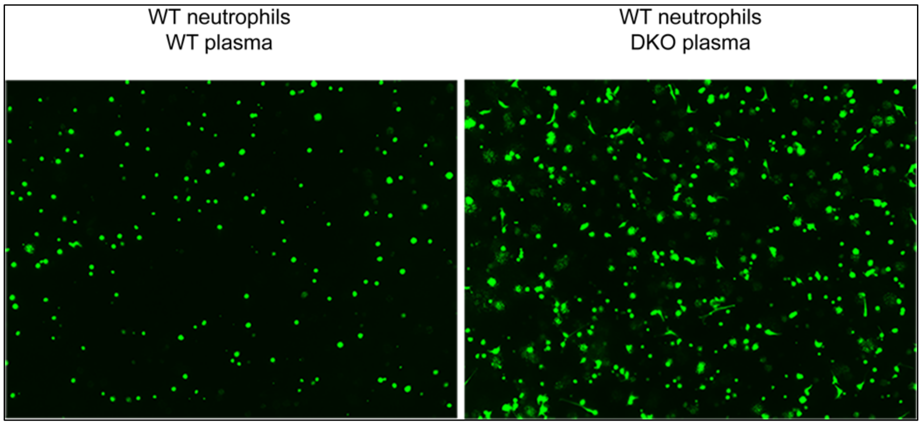

Supplemental Figure 1. Plasma from DKO mice induces NETs in WT mouse neutrophils. Neutrophils from WT mice were isolated and plated into poly-lysine coated wells of a 96-well plate and allowed to adhere at 37°C in 100ul RPMI. After 1hr, the wells were spiked with 10ul plasma from either a WT (left) or a DKO (right) mouse and incubated for 2hrs, and images were captured on a fluorescent microscope after adding Sytox Green (5uM). The WT neutrophils demonstrate increased fluorescence after being exposed to the plasma from a DKO mouse (right) compared to when the same neutrophils are exposed to the plasma of a WT mouse (left). Image captured at 40X magnification.

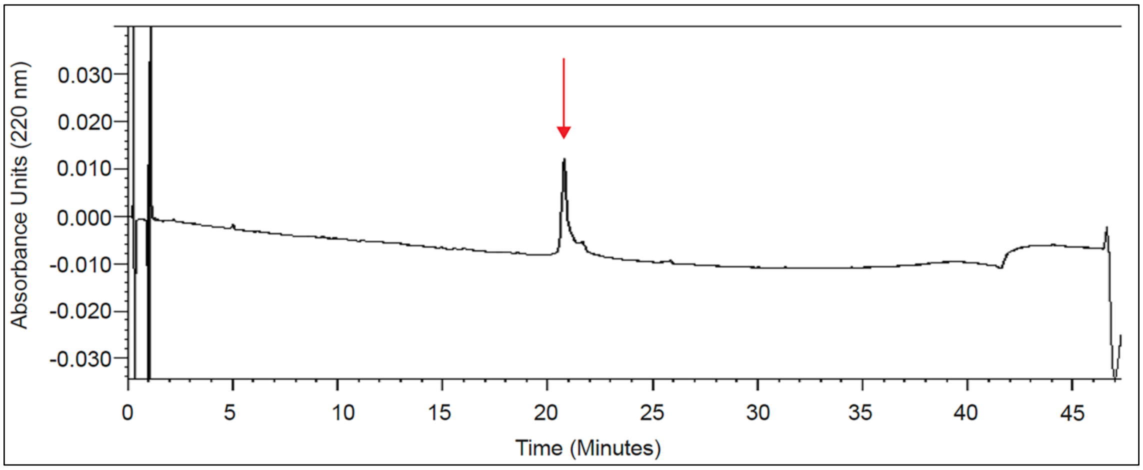

Supplemental Figure 2. HPLC analysis of 1833. LC-PDA analysis performed on 1833 following purification revealed a single major peak with a small trailing peak.

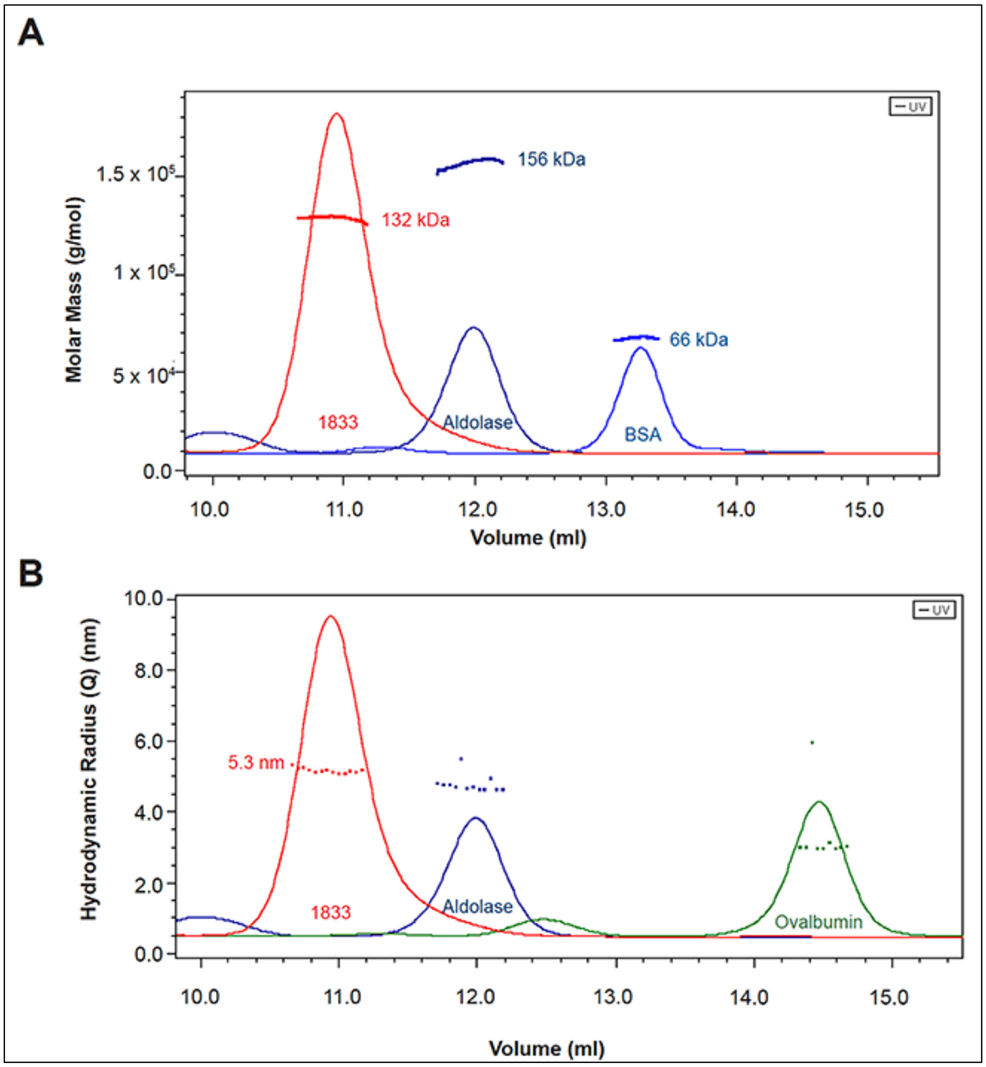

Supplemental Figure 3. Size-exclusion chromatography – light scattering chromatography of 1833. Size-exclusion chromatography – light scattering, undertaken to assess aggregation state and hydrodynamic behavior, revealed that 1833 eluted as a single peak, **(A)** with the predicted molecular weight of a stable dimer at two separate concentrations of sample loading. The eluting sample contained ≈119 kDa of protein and ≈13kDa of sugars for a total molecular weight of 132 kDa and exhibited **(B)** a hydrodynamic radius of 5.3nm

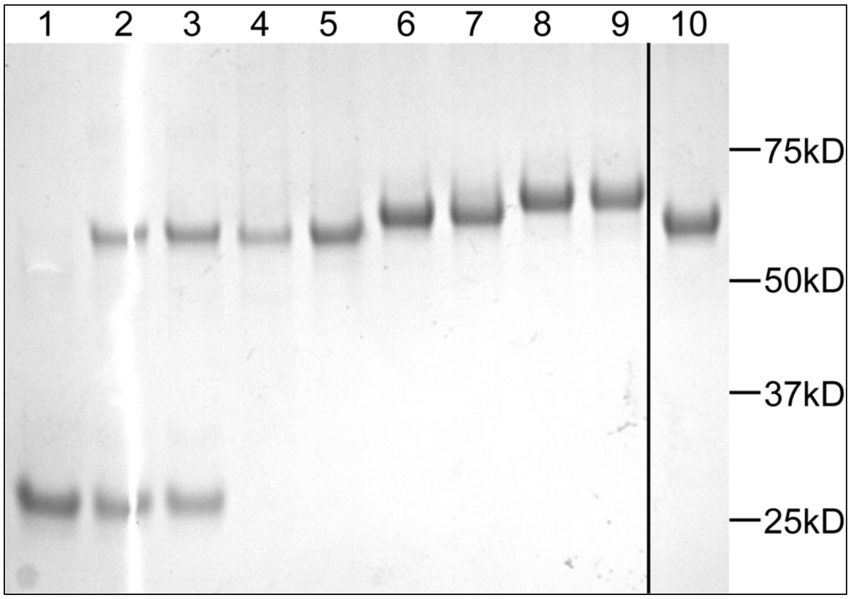

Supplemental Figure 4. Coomassie stained gel of sample CHO cell purified proteins used in this study. Lanes 1, 2, and 3 show three unsuccessful attempts to purify full-length mouse DNASE1-IgG1-Fc fusion protein (predicted MW of 58kD) from construct 1171. A cryptic trypsin cleavage site, identified and mutated by a single R290G amino acid substitution, resulted in a well-behaved full-length protein (Lane 4 construct 1585). Lane 5 (construct 1671) with the hyperactive E35R amino acid substitution. Lanes 6 and 7, show two preparations of construct LBme with a mobility shift indicative of the successful N-linked glycosylation of newly engineered glycosylation consensus sites. Similarly, lanes 8 and 9 are two preparations of construct 1689, which also displays a glycosylation induced mobility shift. Lane 10 is human DNASE1-Fc, construct 1833.

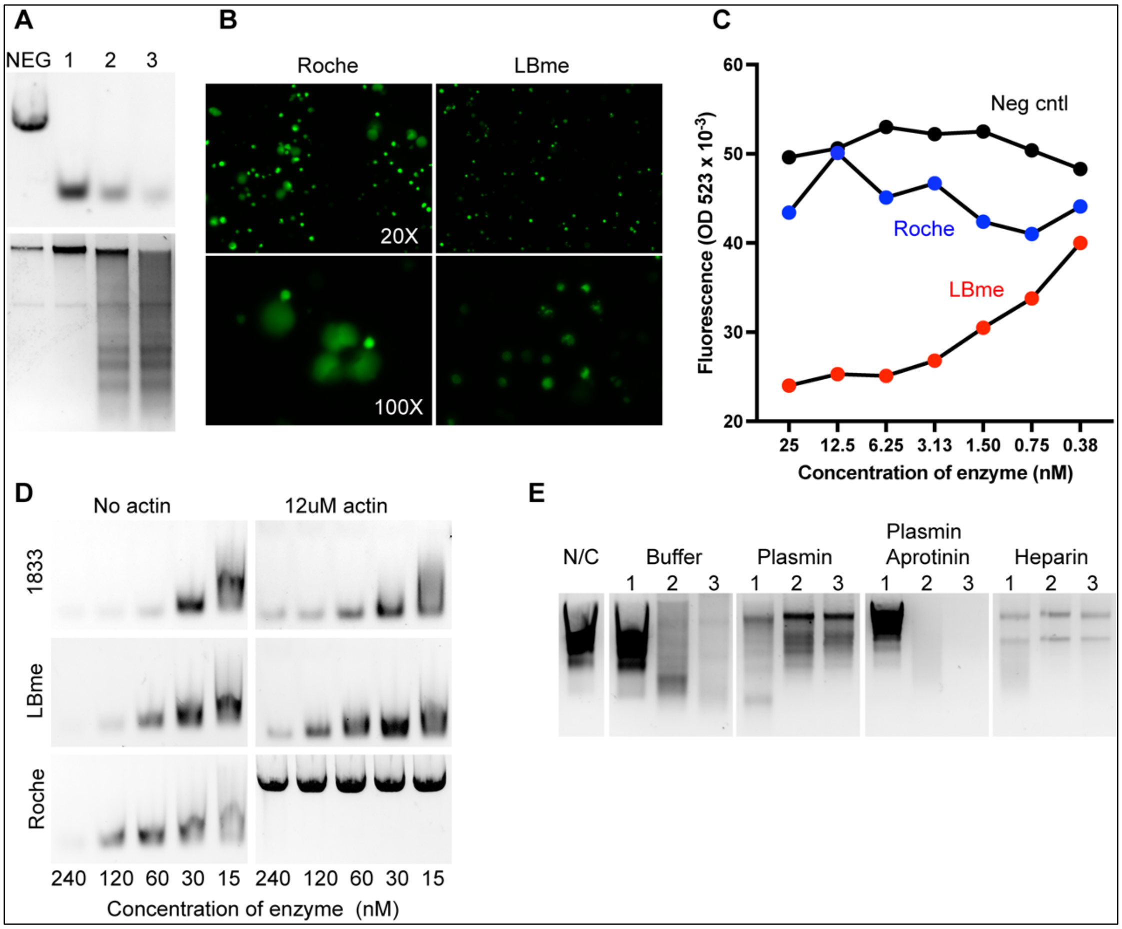

Supplemental Figure 5A-E. Comparison of LBme and human isoform (1833) with commercial DNAse1 (Roche). (A) LBme and 1833 degrade both plasmid (top) and chromatin (bottom) better than the equivalent concentration of commercially available DNASE1 from Roche. NEG: negative control (no DNASE added); lane 1: Roche DNASE1; lane 2: LBme; lane 3: 1833. **(B)** Extruded NETs are efficiently degraded by LBme in contrast to Roche DNASE1. Purified neutrophils were induced to form extracellular traps with 50nM PMA in the presence varying concentrations of biologic (25 to 0.38nM). After 4 hours, 5uM cell impermeable Sytox green was used to visualize and quantitate the amount of NETs remaining. Representative images taken from 6.25nM concentration Roche (Left) or LBme (Right) showing a reduction in diffuse green staining of NETs by LBme with only the fluorescent signal from dead cell nuclei remaining. **(C)** The amount of SYTOX Green signal remaining in each well after NET digestion in the presence of varying concentrations of enzyme was quantitated using a fluorescence microplate reader. **(D)** In the presence of 12uM actin, a known inhibitor of DNASE1, all concentrations of Roche DNASE1 tested were inhibited while both LBme and 1833 demonstrate complete activity in the presence of actin at all concentrations tested. **(E)** The inter-nucleosome cleavage of chromatin by LBme and our lead human DNase1-Fc isoform – 1833 – is not inhibited by heparin (10U/mL) or plasmin (30U/mL), which are known inhibitors of DNASE1L3. Plasmin, a serine protease that can be inhibited by aprotinin, activates the ability of DNASE1 to cleave chromatin. Indeed, in the presence of plasmin all three biologics can digest chromatin but the addition of aprotinin (30U/mL) prevents Roche DNASE1 without affecting the ability of LBme or 1833 to cleave chromatin. Heparin binds to and inactivates DNASE1L3 but also activates the ability of DNASE1 to cleave chromatin by displacing histone in the nucleosome core. In the presence of heparin all three biologics are able to digest chromatin indicating that neither LBme and 1833 are inhibited by heparin.

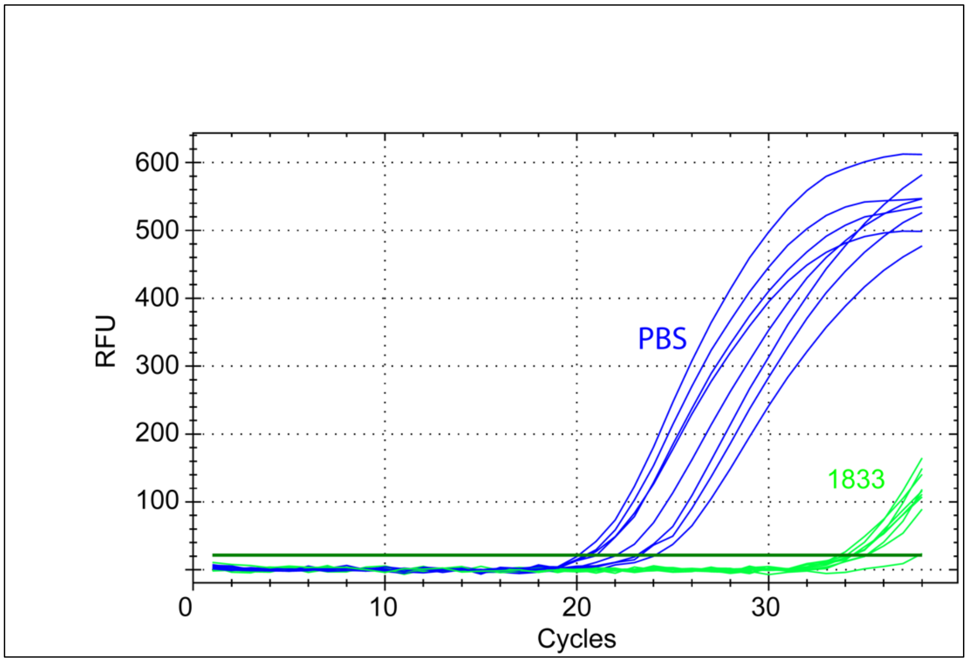

Supplemental Figure 6. Digestion of gDNA by 1833 in the Human whole blood. Whole blood with EDTA from an SLE patient and a HC was spiked with 20mM CaCl2, 20mM MgCl2 to compensate for the EDTA and then 200uM 1833 or PBS was added for a 10 min incubation at 37C. After which the samples were placed on ice and then centrifuged at 1000 xg for 10min at 4C and plasma was transferred to a new tube from which 1ul was used in a qPCR reaction with oligos directed at the human LINE-1 element in quadruplicate, as previously describe. Untreated samples are shown in blue and the 1833 treated samples are shifted right in green line. Some Ct values were not obtained because there was not enough signal to cross the threshold.

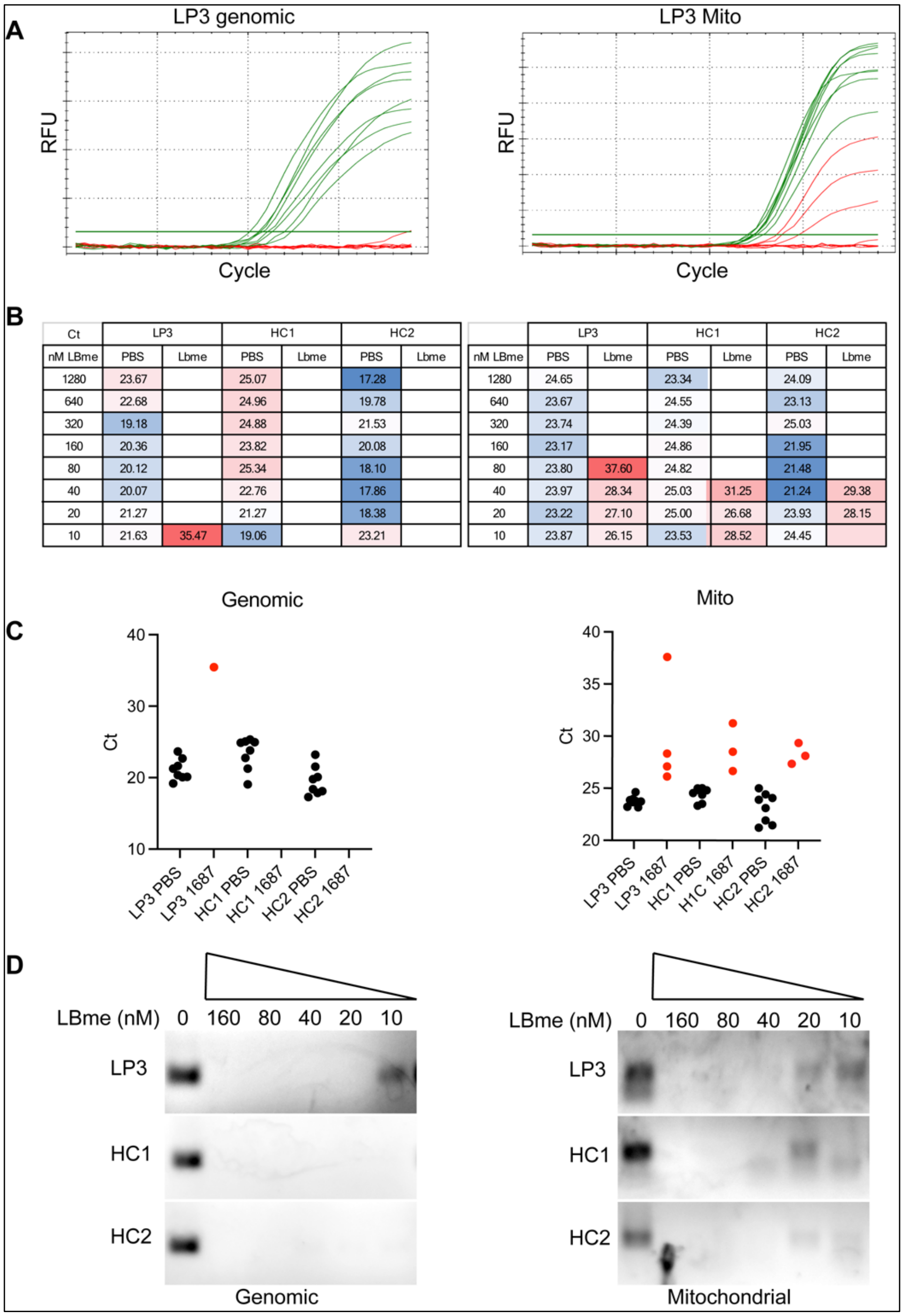

Supplemental Figure 7A-D. Digestion of genomic and mitochondrial DNA in human whole blood of SLE LBme. Blood drawn from a lupus patient (LP3) and two healthy controls (HC1+HC2) and placed in 8-well strips with serial dilutions of LBme or an equivalent volume of PBS. After a 10min incubation the strips were centrifuged at 1000 xg for 5 min at 4C and 1ul of plasma was analyzed by qPCR. The data is presented in 4 formats to illustrate the effectiveness of LBme at digestion cfDNA. **(A)** A representative plot of the Relative Fluorescence Units (RFU) vs the cycle number for Lupus patient #3 (LP3) for both the genomic (left) and mitochondrial (right) profiles. The 8 PBS treated profiles are shown in green and the 8 titrated LBme profiles are shown in red shifted to the right. The horizontal red lines indicate complete digestion of both genomic and mitochondrial DNA by the higher concentrations of LBme. **(B)** The cycle threshold (Ct) data for each reaction with a conditional formatted heat map to illustrate the relative levels of DNA with

blue (low Ct values) predominantly in the PBS treated samples indicating an abundance of DNA and red (high Ct values) predominantly in the LBme digested samples indicating a reduction of DNA. Empty boxes represent a complete absence of detectable DNA. **(C)** The same data plotted in Prism GraphPad with most LBme treated absent (undetectable DNA levels that do not cross the Ct) and those that had detectable DNA shown in red. **(D)** Representative reactions analyzed on a 2% agarose gel Stain with Ethidium Bromide. The genomic amplimer seen without LBme is nearly completely absent in all samples treated with LBme with the exception of LP3 at the lowest LBme concentration. Meanwhile the mitochondrial amplimer is reduced at most concentrations of LBme but some is still detected at the lower concentrations of LBme tested.

Supplemental References

1. Yang H, Wang H, and Jaenisch R. Generating genetically modified mice using CRISPR/Cas-mediated genome engineering. *Nature Protocols.* 2014;9(8):1956-68.
2. Price NL, Rotllan N, Zhang X, Canfrán-Duque A, Nottoli T, Suarez Y, et al. Specific Disruption of Abca1 Targeting Largely Mimics the Effects of miR-33 Knockout on Macrophage Cholesterol Efflux and Atherosclerotic Plaque Development. *Circulation Research.* 2019;124(6):874-80.
3. Quadros RM, Miura H, Harms DW, Akatsuka H, Sato T, Aida T, et al. Easi-CRISPR: a robust method for one-step generation of mice carrying conditional and insertion alleles using long ssDNA donors and CRISPR ribonucleoproteins. *Genome Biology.* 2017;18(1):92.
4. Concordet JP, and Haeussler M. CRISPOR: intuitive guide selection for CRISPR/Cas9 genome editing experiments and screens. *Nucleic Acids Res.* 2018;46(W1):W242-W5.
5. Nagy. *Manipulating the Mouse Embryo: a Laboratory Manual.* Cold Spring Harbor, NY: Cold Spring Harbor Laboratory Press; 2003.
6. Swamydas M, Luo Y, Dorf ME, Lionakis MS. Isolation of Mouse Neutrophils. Curr Protoc Immunol. 2015 Aug 3;110:3.20.1-3.20.15

Table 1: Mouse Dnase1 oligo sequences (11 pairs of oligos).

Oligo ID	Dnase1 oligos	Notes	Directionality
381	ggatccaccatgcggtacacagggctaatgg	Dnase I	Sense
382	agcctcgagattttctgagtgtcacc	Dnase I	Anti-Sense
385	ccacaacacacgtgaccttaggctccagagtaatatagagcacatccttgggctttggg	T318Y, T322E	Sense
386	cccaaagcccaaggatgtgctctatattactctggagcctaaggtcacgtgtgttgtgg	T318Y, T322E	Anti-Sense
416	gtagcattggacatcttagtcctcccaaaagtccgaatgttgaa	E35R	Sense
417	ttcaacattcggacttttgggaggactaagatgtccaatgctac	E35R	Anti-Sense
522	ggcttacaaccagatccaaccatggtgctcgag	R290G	Sense
523	ctcgagcaccatggttggatctggttgtaagcc	R290G	Anti-Sense
526	cctgacacctaccgctatgtaaagagtgagccgctgggc	V89K	Sense
527	gcccagcggctcactctttacatagcggtaggtgtcagg	V89K	Anti-Sense
528	gggaaaagaacttaacaatgaatggctctctgctgaaggtgt	A136F	Sense
529	acaccttcagcagagagccattcattgttaagttcttttccc	A136F	Anti-Sense
536	gcttcagccagctggttggaaagattgtattctgcttggaaatcaaaag	G262N	Sense
537	cttttgatttccaagcagaatacaatctttccaaccagctggctgaagc	G262N	Anti-Sense
538	cactgatggcttcagccgtctggttggaaagtccgt	L267T	Sense
539	acggactttccaaccagacggctgaagccatcagtg	L267T	Anti-Sense
574	cttctgggactgtacttatgctaggcttactaccagatccaaccat	C293S, C296S	Sense
575	atggttggatctggtagtaagcctagcataagtacagtcccagaag	C293S, C296S	Anti-Sense
540	gatagctgtccagaatagacgtctggttaggcctgtacacaaaaaggt	D109N, V111T	Sense
541	acctttttgtgtacaggcctaaccagacgtctattctggacagctatc	D109N, V111T	Anti-Sense
542	cccagcggctcactgacattatagcggtaggtgtcagg	V88N	Sense
543	cctgacacctaccgctataatgtcagtgagccgctggg	V88N	Anti-Sense

Table 2: Mouse Dnase1L3 oligo sequences (17 pairs of oligos)**.**

Oligo ID	Dnase1L3 Oligos	Notes	Directionality
459	gaattcaccatgtccctgcacccagc	Dnase1L3	Sense
460	gctagcttaggagcgattgcctttttttctctt	Dnase1L3	Anti-Sense
506	ctctttttgagagaaacagaagatctgttgttggtgaaggcccttgaag	Delete NLS	Sense
507	cttcaagggccttcaccaacaacagatcttctgtttctctcaaaaagag	Delete NLS	Anti-Sense
532	tgctctttgtacgtctttcttccaagtcgagaactaatca	N101K	Sense
533	tgattagttctcgacttggaagaaagacgtacaaagagca	N101K	Anti-Sense
534	atggttttccttcttgctccttccaaaggacctcacattg	A38R	Sense
535	caatgtgaggtcctttggaaggagcaagaaggaaaaccat	A38R	Anti-Sense
544	ccgtggttccccgttccaatggcacctttgactttcagaaagc	S257N, V259T	Sense
545	gctttctgaaagtcaaaggtgccattggaacggggaaccacgg	S257N, V259T	Anti-Sense
546	gctcgaggagcgagtgccatttttttctctttttgagagaaacag	K306N, N308T	Sense
547	ctgtttctctcaaaaagagaaaaaaatggcactcgctcctcgagc	K306N, N308T	Anti-Sense
548	cacaatgatatccatggccgtatggttttccttcttgctcgctccaaag	E45T	Sense
549	ctttggagcgagcaagaaggaaaaccatacggccatggatatcattgtg	E45T	Anti-Sense
550	catcagcatgggacaggtgttgttgctgctgtc	I72T	Sense
551	gacagcagcaacaacacctgtcccatgctgatg	I72T	Anti-Sense
552	tttcttccaagtcgagaactaatcgtatagttgtatgttgtgcttcttcg	V93T	Sense
553	cgaagaagcacaacatacaactatacgattagttctcgacttggaagaaa	V93T	Anti-Sense
554	cttcttgaccgtagtgtcattttggtccccaatcagccaa	E226N	Sense
555	ttggctgattggggaccaaaatgacactacggtcaagaag	E226N	Anti-Sense
556	cctggaaaacacgtctgtgtcattatcctgatagtcatggtagtgg	G130N	Sense
557	ccactaccatgactatcaggataatgacacagacgtgttttccagg	G130N	Anti-Sense
558	cacaaagggctccctggaaaacgtgtcattgtctccatcctgatagtcatg	T132N, V134T	Sense
559	catgactatcaggatggagacaatgacacgttttccagggagccctttgtg	T132N, V134T	Anti-Sense
568	catgggacagatgttgttgtggctgtccttgatttccatc	S69H	Sense
569	gatggaaatcaaggacagccacaacaacatctgtcccatg	S69H	Anti-Sense
752	ccatggtgctcgaggagtgattgcctttttttctc	R309H	Sense
753	gagaaaaaaaggcaatcactcctcgagcaccatgg	R309H	Anti-Sense
754	cgaggagcgattgcctttttttttctttttgagagaaacagattt	R304K	Sense
755	aaatctgtttctctcaaaaagaaaaaaaaaggcaatcgctcctcg	R304K	Anti-Sense
756	ctttttgagagaaacagatttttttgctgttggtgaaggcccttgaag	N295S, R296K	Sense
757	cttcaagggccttcaccaacagcaaaaaaatctgtttctctcaaaaag	N295S, R296K	Anti-Sense
574	cttctgggactgtacttatgctaggcttactaccagatccaaccat	C322S, C324S	Sense
575	atggttggatctggtagtaagcctagcataagtacagtcccagaag	C322S, C324S	Anti-Sense

Table 3: Human DNAse1 oligo sequences (10 pairs of oligos).

Oligo ID	DNASE1 oligos	Notes	Directionality
615	ggtgtgggtcttgttggaggagcccttca	D286N	Sense
616	tgaagggctcctccaacaagacccacacc	D286N	Anti-Sense
617	ggagaggtgtggttcttgtcggaggagcc	T288N	Sense
618	ggctcctccgacaagaaccacacctctcc	T288N	Anti-Sense
619	ggcctgagccgtctgattagacaggccgtaggcggct	D265N, L267T	Sense
620	agccgcctacggcctgtctaatcagacggctcaggcc	D265N, L267T	Anti-Sense
621	aaaggcagggtagaattaggcaccacagcaccctgg	D250N, A252T	Sense
622	ccagggtgctgtggtgcctaattctaccctgccttt	D250N, A252T	Anti-Sense
623	aggccgtaggcggccgtgaagttgaaaggcagggca	Q258T	Sense
624	tgccctgcctttcaacttcacggccgcctacggcct	Q258T	Anti-Sense
625	ccactggaaggtaggattggtccacagccggata	S211N	Sense
626	tatccggctgtggaccaatcctaccttccagtgg	S211N	Anti-Sense
629	ccacggcggacacattgtcaggccggtac	Q110N	Sense
630	gtaccggcctgacaatgtgtccgccgtgg	Q110N	Anti-Sense
631	ggtgtcgttgcggcagggctcgc	G127R	Sense
632	gcgagccctgccgcaacgacacc	G127R	Anti-Sense
788	cagctgatcagacaggttgtaggcggcttggaag	G262N	Sense
789	cttccaagccgcctacaacctgtctgatcagctg	G262N	Anti-Sense
790	ctttctttgtaggaatttctgcccaggggctcaga	K96N	Sense
791	tctgagcccctgggcagaaattcctacaaagaaag	K96N	Anti-Sense

Table 4: Human DNAse1L3 oligo sequences (8 pairs of oligos).

Oligo ID	DNASE1L3 Oligos	Notes	Directionality
599	tcacgatcacgtccgtggcgttcttgtcctc	M42T	Sense
600	gaggacaagaacgccacggacgtgatcgtga	M42T	Anti-Sense
601	ccagcctggaggagatcgtgtagttgtaggtgatgc	V88T	Sense
602	gcatcacctacaactacacgatctcctccaggctgg	V88T	Anti-Sense
603	aaaggctcgcgggagaacgtatcgttgtcgccatcctggtagtc	A127N, V129T	Sense
604	gactaccaggatggcgacaacgatacgttctcccgcgagccttt	A127N, V129T	Anti-Sense
605	gcgtactgctctttggtggtattgcggcccagcctg	K96N, Y98T	Sense
606	caggctgggccgcaataccaccaaagagcagtacgc	K96N, Y98T	Anti-Sense
607	ctcctccaggctgggcaacaagacctacaaagag	R95N	Sense
608	ctctttgtaggtcttgttgcccagcctggaggag	R95N	Anti-Sense
609	tcctcttgcttagactcgccgaaggaccgcac	R33E	Sense
610	gtgcggtccttcggcgagtctaagcaagagga	R33E	Anti-Sense
611	ttcttcacggtggtatcattttggtcgccgatcagcc	E221N	Sense
612	ggctgatcggcgaccaaaatgataccaccgtgaagaa	E221N	Anti-Sense
613	gccttttggaagtcgaacgtggagttggacttgggcac	V254T	Sense
614	gtgcccaagtccaactccacgttcgacttccaaaaggc	V254T	Anti-Sense
